# Thiram, an inhibitor of 11ß-hydroxysteroid dehydrogenase type 2, enhances the inhibitory effects of hydrocortisone in the treatment of osteosarcoma through Wnt/β-catenin pathway

**DOI:** 10.1186/s40360-023-00655-0

**Published:** 2023-03-28

**Authors:** You Zhang, Nanjing Li, He Li, Maojia Chen, Wei Jiang, Wenhao Guo

**Affiliations:** 1 Clinical Translational Innovation Center/Molecular Medicine Research Center, West China Hospital, Sichuan Univicity, Chengdu, Sichuan Province 610041 People’s Republic of China; 2 Division of of Radiotherapy, Cancer Center,West China Hospital, Sichuan University, Chengdu, Sichuan Province 610041 People’s Republic of China; 3 West China School of Basic Medical Sciences & Forensic Medicine, Sichuan University, Chengdu, Sichuan Province 610044 People’s Republic of China; 4 Animal Experiment Center, West China Hospital, Sichuan University, Chengdu, 610000 People’s Republic of China; 5 Department of Abdominal Oncology, Cancer Center and State Key Laboratory of Biotherapy, Medical School, West China Hospital, Sichuan University, No. 37, Guoxue Road, Chengdu, Sichuan Province 610041 People’s Republic of China

**Keywords:** 11ß-hydroxysteroid dehydrogenase type 2, Hydrocortisone, Thiram, Osteosarcoma, Wnt/β-catenin pathway

## Abstract

**Background:**

The anti-osteosarcoma effects of hydrocortisone and thiram, an inhibitor of type 2 11ß-hydroxysteroid dehydrogenase (11HSD2), have not been reported. The purpose of this study was to investigate the effects of hydrocortisone alone or the combination of hydrocortisone with thiram on osteosarcoma and the molecular mechanism, and determine whether they can be as new therapeutic agents for osteosarcoma.

**Methods:**

Normal bone cells and osteosarcoma cells were treated with hydrocortisone or thiram alone or in combination. The cell proliferation, migration, cell cycle and apoptosis were detected by using CCK8 assay, wound healing assay, and flow cytometry, respectively. An osteosarcoma mouse model was established. The effect of drugs on osteosarcoma in *vivo* was assessed by measuring tumor volume. Transcriptome sequencing, bioinformatics analysis, RT–qPCR, Western blotting (WB), enzymelinked immunosorbent assay (ELISA) and siRNA transfection were performed to determine the molecular mechanisms.

**Results:**

Hydrocortisone inhibited the proliferation and migration, and induced apoptosis and cell cycle arrest of osteosarcoma cells in *vitro*. Hydrocortisone also reduced the volume of osteosarcoma in mice in *vivo*. Mechanistically, hydrocortisone decreased the levels of Wnt/β-catenin pathway-associated proteins, and induced the expression of glucocorticoid receptor α (GCR), CCAAT enhancer-binding protein β (C/EBP-beta) and 11HSD2, resulting in a hydrocortisone resistance loop. Thiram inhibited the activity of the 11HSD2 enzyme, the combination of thiram and hydrocortisone further enhanced the inhibition of osteosarcoma through Wnt/β-catenin pathway.

**Conclusions:**

Hydrocortisone inhibits osteosarcoma through the Wnt/β-catenin pathway. Thiram inhibits 11HSD2 enzyme activity, reducing hydrocortisone inactivation and promoting the effect of hydrocortisone through the same pathway.

**Supplementary Information:**

The online version contains supplementary material available at 10.1186/s40360-023-00655-0.

## Introduction

Osteosarcoma is a primary malignant bone tumor that metastasizes easily and is most prevalent in children and young adults under 20 years of age [1]. In recent years, some glucocorticoids have been reported to inhibit osteosarcoma cell proliferation [2] [3], which provides an idea for the treatment of osteosarcoma with glucocorticoid drugs. Hydrocortisone (HC) is a synthetic glucocorticoid mainly used in the clinical treatment of adrenocortical hypofunction [4]. However, the effect of HC on osteosarcoma and the glucocorticoid resistance of osteosarcoma cells have not been reported.

To date, the main known cause of glucocorticoid resistance is the disruption of glucocorticoid receptor α (GCR). However, because 11β-hydroxysteroid dehydrogenase type 2 (11HSD2) can inactivate glucocorticoids in tissues [5, 6], 11HSD2 may also be one of the causes of resistance to GC. Thiram, a fungicide with moderate toxicities (LD50-375 mg/kg bw (rat), 1 g/kg bw (mice), 210 mg/kg bw (rabbits)) [7], can inhibit the enzyme activity of 11HSD2 in rats and humans [8, 9], and the antitumor effect of thiram combined with HC and its effect on HC drug resistance have not been reported.

To clarify the inhibitory effect of HC on osteosarcoma and the drug resistance and molecular mechanism generated by HC on osteosarcoma, we planned to treat osteosarcoma cells and osteosarcoma model mice with HC, thiram and si-11HSD2 separately and in combination. To determine whether the combination of thiram with HC as an inhibitor of 11HSD2 can enhance the anti-osteosarcoma effect of HC, the proliferation and migration of tumor cells and tumor volume in model mice before and after drug administration were analyzed, and the mechanism was studied by investigating apoptosis, the cell cycle and protein signaling pathways.

HC is mainly used for adrenal hypocorticosis and hypopituitarism [10] in clinic. Currently, there is no study on the effect of HC on osteosarcoma, no study on reducing hydrocortisone resistance, and no study on the synergistic effect of enzyme inhibitors combined with HC. If the problem can be solved, it will provide new drugs for the treatment of osteosarcoma, provide more ideas for the clinical application of HC, and bring good news to patients with other diseases affected by HC drug resistance.

## Materials and methods

### Materials

Antibodies against residues 261–405 of human 11β-HSD2 (Thermo Fisher, CA, USA) and the rest of the antibodies were purchased from Beyotime Biotechnology (Beyotime, Beijing, China). Nuclear and cytoplasmic protein extraction Kit (Beyotime Biotechnology, Beijing, China), protein detection kit (Beyotime Biotechnology, Beijing, China), ECL detection substrate kits (GE Healthcare, Pittsburgh, PA), and MMP-7 ELISA kits (Sangon Biotech, Shanghai, China) were used in the present study. The chemicals used were thiram (PESTANAL, > 99%, Sigma) and hydrocortisone (HC, Baiyunshan Pharmaceutical, China). All chemicals were analytical grade.

### Cell culture and compounds

The K7M2-wt, Saos-2, hFOB1.19, MC3T3-E1 Subclone 14 and MG63 cell lines were obtained from the Cell Culture Center, Shanghai Institutes for Biological Sciences, Chinese Academy of Sciences (Shanghai, People’s Republic of China). Cells were cultured at 37 °C with 5% CO_2_ saturation in high-glucose DMEM (HyClone, GE, USA) or F12-MEM containing 10% heat-inactivated fetal bovine serum (Gibco, Thermo Scientific, USA), penicillin 100 IU/ml (Invitrogen, Carlsbad, CA, USA) and streptomycin 100 µg/ml (Gibco, Thermo Scientific, USA), and the fluid was changed every 48 h. In addition, 0.6 mg/mL G418 was added to cultured hFOB1.19 cells. The experimental concentrations of HC and thiram were 625 µM and 1.5 µM, respectively.

### Western blotting

Western blot analysis was performed following the method described previously [11]. For all WBs, a 1:2000 dilution was used for the primary antibodies (Thermo Fisher Scientific, USA), and the species-specific secondary IgG was diluted to 1:4000 (ZSGB-BIO, Beijing, China).

### Reverse transcription quantitative-polymerase chain reaction (RT–qPCR) analysis

Total cellular RNA was extracted using TRIzol reagent (Invitrogen) according to the manufacturer's instructions. RNA was reverse transcribed to produce cDNA, and real-time PCR amplification was performed as described previously [12]. The specific primer pairs (Shanghai Sangong Co., Ltd., Shanghai, China) were as follows: beta-actin forward (internal reference), 5′-GATGCTGGTGCTGAGTATGACG-3′ and reverse 5′-GTGGTGCAGGATGCATTGCTCTGA-3′; 11HSD2 forward, 5′- TGCTGGCTCTTCCTAGA -3′ and reverse, 5′-ATGGCATCTACGGCTGGGCT-3′.

### Transcriptome sequencing and bioinformatics analysis

The samples were sent to Shanghai Meggie Biotechnology Co., Ltd. for subsequent transcriptome sequencing experiments. The mRNA expression profiles between the control and HC groups were compared and analyzed, including differential gene expression screening, Gene Ontology (GO) enrichment analysis and pathway analysis based on the Kyoto Encyclopedia of Genes and Genomes (KEGG) database [13, 14]. A pathway map was plotted based on the bioinformatics analysis and WB results.

### Transfection

Lipofectamine 2000 was used to perform transfection according to the manufacturer's instructions [15]. Briefly, cells were cultured to 50% confluence and resuspended in serum-free medium. Small interfering RNA (siRNA) specific for 11HSD2 (Sangon Biotech, Shanghai, China) and Lipofectamine 2000 were diluted, mixed and incubated for 20 min at room temperature, followed by addition to the cell suspension. After incubation at 37 °C for 8 h, the medium was replaced with normal serum-containing medium. Cells were then cultured for 48 h prior to being subjected to the following assays. MG63 and Saos-2 cells transfected with si-11HSD2 were treated with drugs for 48 h. The interference sequence of 11HSD2 was 5′- GCGUGCUAGAGUUCACCAATT-3′ (forward) and 5′-UUGGUGAACUCUAGCACGCTT-3′ (reverse).

### Enzyme-linked immunosorbent assay (ELISA) for measuring matrix metalloproteinase 7 concentrations

The amount of MMP7 released from the osteosarcoma cells was determined using specific ELISA kits (D711196, Sangon Biotech) according to the protocols provided by the manufacturer. Briefly, osteosarcoma cells (15 × 10^4^/well) were plated in 6-well plates and/or treated with drugs for 48 h. Cell culture solution was collected and added to an ELISA plate, and the plate was washed after 90 min in a 37 °C bath. Then, biotin-conjugated antibody was added, and the plate was washed after a 60-min warm bath at 37 °C. Then, 100 μl/well HRP-conjugated streptavidin was added to the bath at 37 °C for 30 min. After the bath, 90 µl substrate reagents were added to test the absorbability at 450 nm. The standard curve was calculated according to the absorption value, and the protein concentration was calculated according to the formula of the standard curve.

### Cell viability assay

Osteosarcoma cells were seeded in 96-well plates at a density of 5,000 cells per well. They were treated with different concentrations of HC and/or thiram for 48 h, and cell viability was measured by CCK-8 assay as described previously [16].

### Apoptosis assay

The apoptotic cell percentage was quantified by an Annexin V-FITC/PI detection kit (FXP145, 4abio Biotech, China) as described previously [17]. Cells were cultured in 6-well plates at a density of 1.5 × 10^5^ cells per well. The cells were treated with HC and/or thiram for 48 h. After removal of the medium, cells were digested with 0.25% trypsin without ethylenediaminetetraacetic acid, centrifuged at 110 g for 5 min, and resuspended in 400 μl binding buffer (0.1 M HEPES/NaOH, 1.4 M NaCl, 25 mM CaCl_2_). After the addition of 5 μl Annexin V-FITC and 5 μl propidium iodide (PI), the cells were allowed to react for 5 min at room temperature in the dark and then analyzed by flow cytometry.

### Cell cycle distribution

Cells were cultured in 6-well plates at a density of 2 × 10^5^ cells per well, incubated for 24 h, and treated with HC and/or thiram for 48 h. The cells were then harvested with trypsin without ethylenediaminetetraacetic acid, washed with cold PBS, and fixed with precooled 75% ethanol overnight at 4 °C. The cells were washed twice with PBS, centrifuged and treated with RNase A and propidium iodide (PI) for 30 min at 37 °C in the dark. Finally, the stained cells were analyzed by a flow cytometer (FXP0211, 4abio Biotech, China).

### Animal experiments

Four- to five-week-old BALB/c mice (female, 16 ± 1 g) were supplied by the Experimental Animal Center of Academy of Military Medical Sciences (Beijing, China). The animals were housed individually and kept in the vivarium in a temperature-controlled environment (22 °C) and a 12 h light/dark cycle (lights on at 7:00 A.M.). Twenty-eight mice were randomly assigned to four groups, each of which was subcutaneously injected with 100 µl of cell suspension (containing 4_˟_10^6^ K7M2-wt cells) [18]. The experimental concentrations of HC and thiram were 5.56 mg/kg and 2 µg/kg, respectively. Twenty microliters of each drug was intraperitoneally injected every other day. All animals were euthanized by cervical dislocation, tumors were harvested on the 10th day, and further experiments were carried out. All experimental procedures took place during the light cycle, between 9:30 A.M. and 5:00 P.M. This experiment was approved by the committee of The West China Hospital of Science and Technology, and humanistic care was obtained under the suggestion of the Principles of Laboratory Animal Care and Guide for the Care and Use of Laboratory Animals.

### Wound healing assay

Wound healing assays were performed following a method described previously [19]. The cells were seeded into a six-well plate containing the culture medium. After 24 h, the cells were wounded with a p200 tip, washed with PBS and then treated with HC and/or thiram. At 48 h, pictures were taken to analyze the migration capacity of cells.

### 11β-HSD2 activity assay

11HSD2 activity was determined by measuring the conversion of cortisol to cortisone [20] or the conversion of corticosterone to 11-dehydrocorticosterone [21] after treatment of the osteosarcoma cells with HC and/or thiram for 48 h. After washing, cell lysates were collected before and after treatment. Cortisone/cortisol (Cortisone/Cortisol Assay Kit, H205/H094, Nanjing Jiancheng Bioengineering Institute, China) and corticosterone (Corticosterone ELISA Kit, D721183, Sangon Biotech, China) levels were then measured with enzyme immunoassay kits. The 11-dehydrocorticosterone assay was performed as described previously [21].

### Statistical analysis

Values are presented as the mean ± SD. The difference between groups was tested by Student’s t test for two groups or ANOVA for multiple groups. Values of *p* < 0.05 were regarded as a significant difference.

## Results

### The combination of HC and thiram decreased proliferation in vivo and in vitro

**Fig.1:**
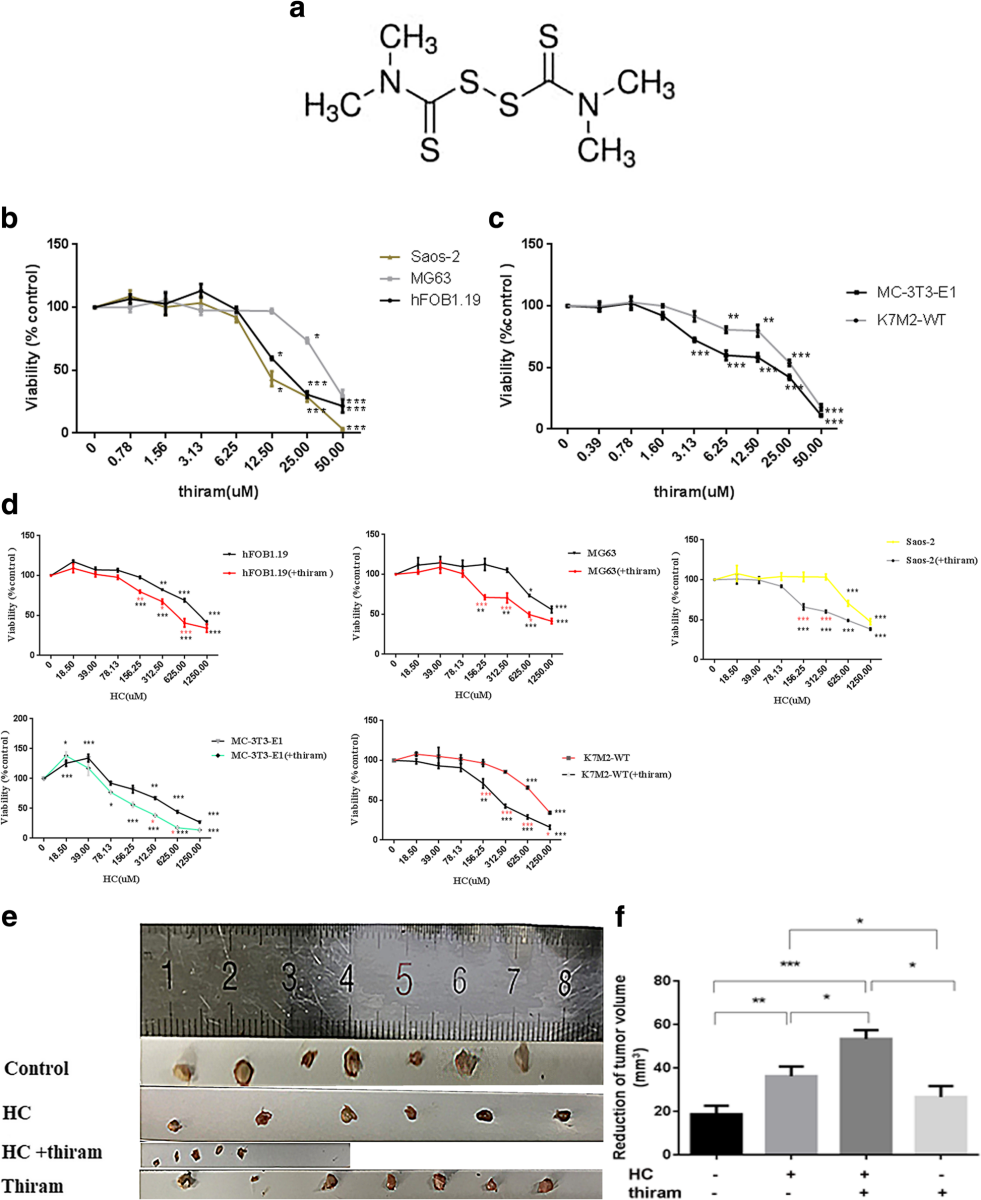
Effects of HC and thiram on osteosarcoma in *vitro* and in mice. **A** The molecular structure of thiram. **B** Effect of thiram on normal human osteocytes and human osteosarcoma cells. **C** Effect of thiram on normal mouse osteocytes and mouse osteosarcoma cells. **D** The combination of HC and thiram inhibited the proliferation of osteosarcoma cells more than HC alone. The red * is the comparison of HC and HC combined with thiram. The black * is the comparison between the HC and control groups. **E** The obtained tumor tissues in each group (the tumors of one mouse in the HC treatment group and two mice in the HC combined with thiram group kept decreasing and then disappeared during drug treatment, so their tumors could not be collected). **F** The tumor shrinkage after drug treatment. B-D Data are presented as the mean ± SE (*n* = 3). * *p* < 0.05,** *p* < 0.01,*** *p* < 0.001

The molecular structure of thiram is shown in Fig. 1A. hFOB1.19 (human osteocytes), MC3T3-E1 Subclone 14 (mouse osteocytes), MG63 (human osteosarcoma cells), Saos-2 (human osteosarcoma cells) and K7M2-WT (mouse osteosarcoma cells) cells were treated with thiram for 48 h. The cytotoxicity of thiram was investigated, and the results are shown in Fig. 1B and Fig. 1C. The results showed that the cell viability of all cells did not change significantly when thiram was lower than 6.25 µM. The viabilities of hFOB1.19 and Saos-2 cells were significantly decreased when the concentration of thiram was greater than 12.5 µM, while that of MG63 cells was significantly decreased when the concentration of thiram was 25 µM. In mouse cells, no significant effect on viability was found when the concentration of thiram was lower than 1.6 µM. Combined with the results of Fig. 1B and Fig. 1C, 1.5 μM thiram did not produce significant toxicity to normal osteocytes and osteosarcoma cells, so this concentration was selected for further study.

The cells were divided into the HC treatment group and HC combined with thiram group, which were administered for 48 h, and then cell viability was detected by a CCK-8 kit. The results are shown in Fig. 1D. The growth of normal cells and tumor cells was significantly inhibited by 312.5 µM and 625 µM HC, respectively, indicating that tumor cells may be resistant to HC. In the combination of HC and thiram experiment, when HC exceeded 156.25 µM, the survival rates of both normal cells and tumor cells began to decrease significantly, indicating that the combination of drugs reduced the concentration of HC used to treat tumor cells.

Then, a mouse tumor model of osteosarcoma was constructed according to the “animal experiments”. On day 5, each tumor grew to 150–200 mm^3^ [22]. The osteosarcoma mice were randomly divided into the control group, HC group, HC and thiram group and thiram group with 7 mice in each group, and drug administration was started. It was observed that the tumors in the control and thiram groups continued to grow but gradually shrank later (tumors shrink in size with time, this phenomenon is often observed in mice with good immunity, which may be due to antitumor immunity [23, 24]). The tumors in the HC group and drug combination group stopped growing and then started shrinking during drug treatment. Ten days later, the mice were sacrificed, the tumor tissue was collected, and the volume was measured, as shown in Fig. 1E. Therefore, the reduction in tumor volume was calculated as the tumor volume on the 5th day minus the tumor volume on day 10. After calculation, the mean tumor reduction was 18.8 mm^3^, 36.3 mm^3^, 53.4 mm^3^ and 23.7 mm^3^ in the control group, HC group, HC combined with thiram group and thiram group, respectively. Therefore, after HC treatment, the tumor volume decreased by 17.5 mm^3^ on average compared with the control group, and thiram combined with HC further reduced the tumor volume by 17.1 mm^3^ compared with the HC group. There was no significant change in tumor volume in the thiram group compared with the control group as shown in Fig. 1F. These results indicated that HC can reduce the tumor volume in mice and that the combination of HC and thiram can further inhibit tumors.

### The combination of HC and thiram decreased the migration of osteosarcoma cells

**Fig. 2:**
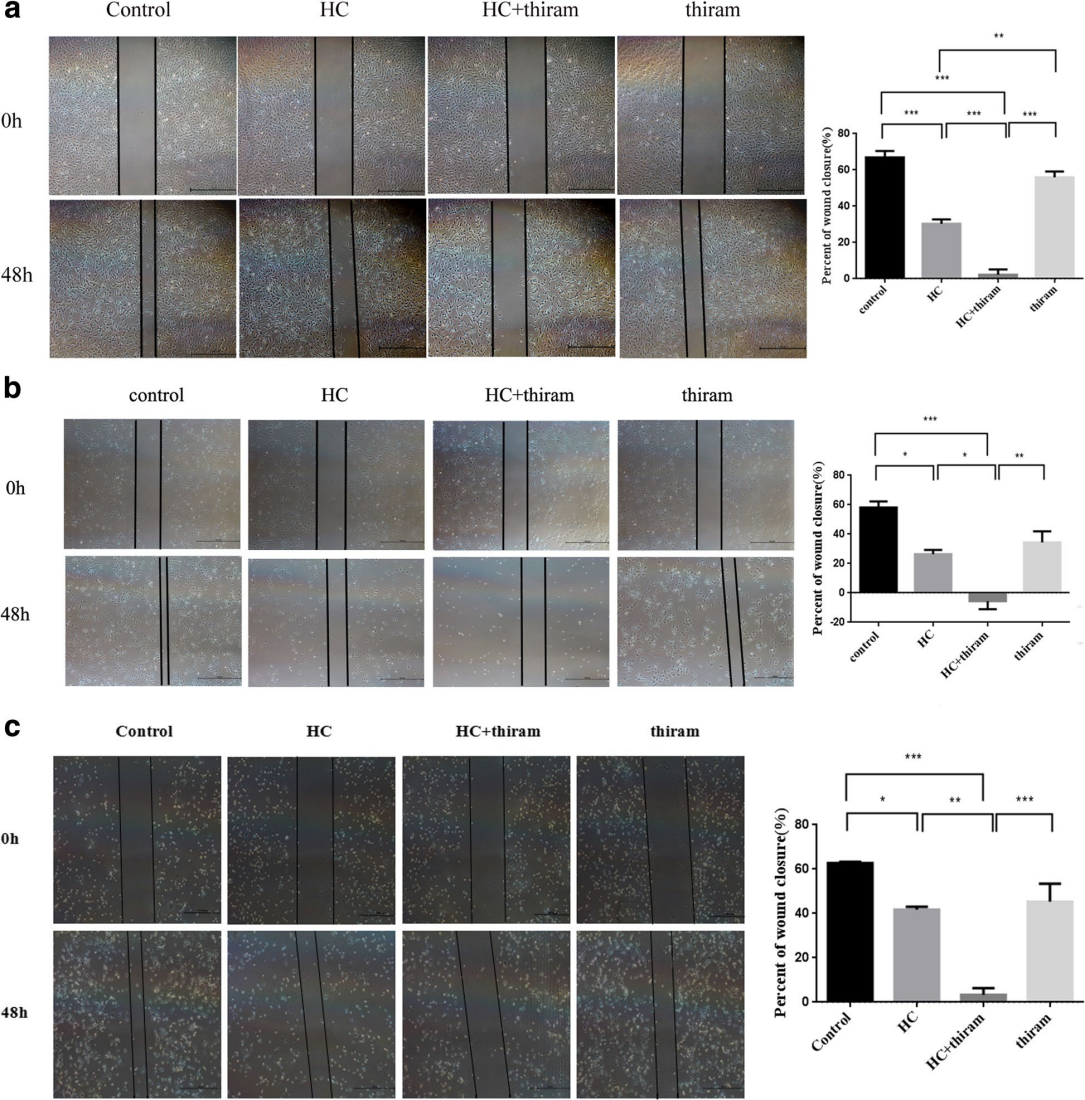
Effects of thiram and HC on the migration of osteosarcoma cells. **A** Effects of drugs on the migration of MG63 cells. **B** Effects of drugs on the migration of Saos-2 cells. **C** Effects of drugs on the migration of K7M2-WT cells. The unhealed area was measured, and the values were organized and shown in the histogram. The initial wound area was used as a 100% control. Scale bars: 100 µM. Data are the mean ± S.E.M. (*n* = 3) and represented as the fold change. *p < 0.05, **p < 0.01, and ***p < 0.001 compared with the control (one-way ANOVA with Tukey’s multiple comparison test)

Figure 2A-C show that the migration distance of osteosarcoma cells treated with HC for 48 h was significantly lower than that of the untreated group, and the migrations of MG63, Saos-2 and K7M2-WT cells were decreased by 36.6%, 31.7% and 21.0% compared with the control group, respectively. The migration of the HC combined with thiram group was further reduced, and the migration of MG63, Saos-2 and K7M2-WT cells was decreased by 28.1%, 32.0% and 38.34%, respectively, compared with that of the HC group. There was no significant difference between the thiram group and the untreated group.

### The combination of HC and thiram promoted apoptosis and cell cycle arrest in osteosarcoma cells

**Fig. 3:**
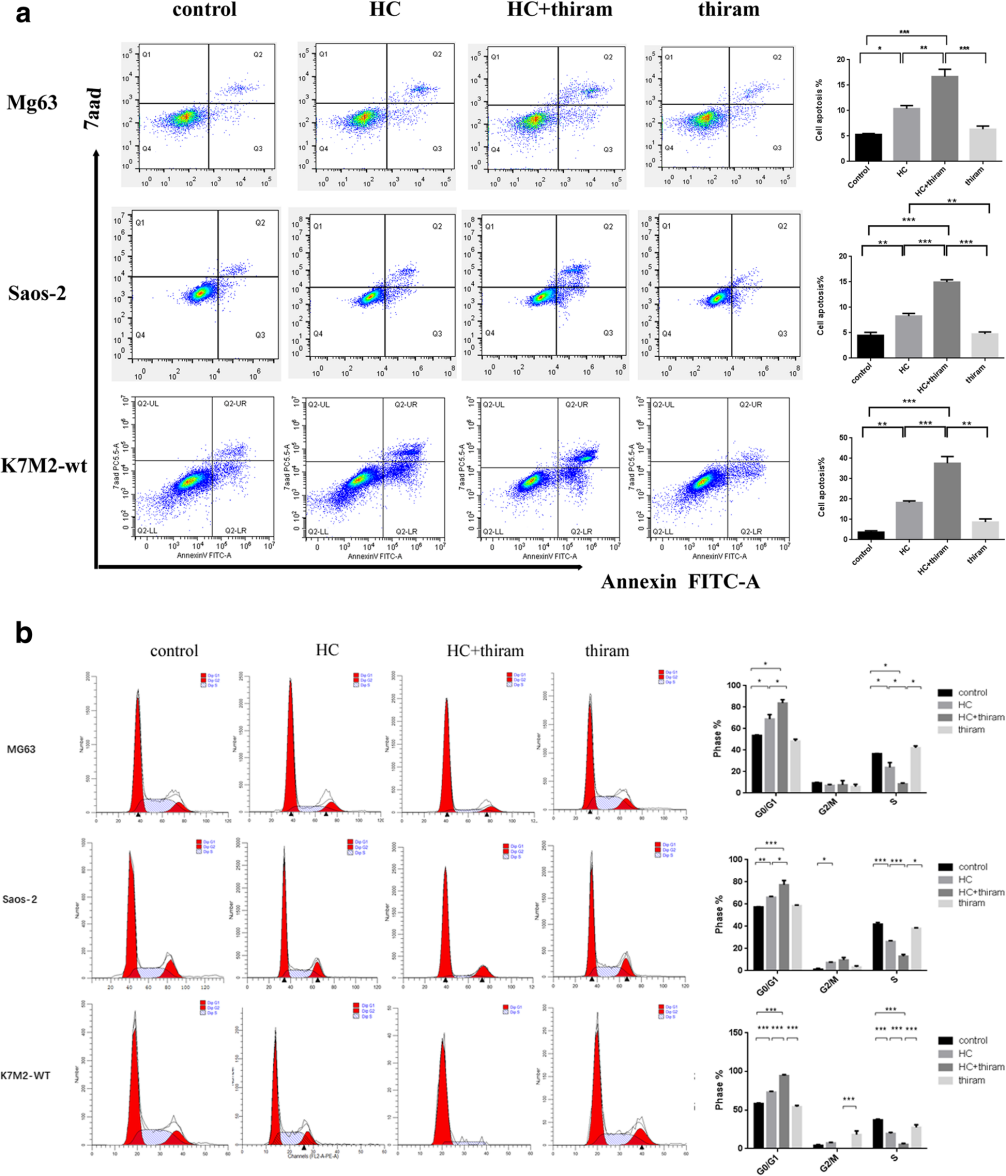
The combination of HC and thiram increased apoptosis and cell cycle arrest in osteosarcoma cells. **A** Apoptosis of osteosarcoma cells by flow cytometry. **B** The cell cycle assay of osteosarcoma cells by flow cytometry. Data are the mean ± S.E.M. (*n* = 3). *, *p* < 0.05, **p < 0.01, ***, *p* < 0.001 compared with the control (one-way ANOVA with Tukey’s multiple comparison test)

After HC treatment, the osteosarcoma cells showed different degrees of apoptosis, and apoptosis was further increased after combined thiram treatment, as shown in Fig. 3A. Compared with the control group, the average apoptosis of Mg63, Saos-2 and K7M2-WT cells in the HC group was increased by 5.1%, 3.8%, and 14.6%, respectively. The average apoptosis rate of the combined treatment group was increased by 6.3, 6.7%, and 19.3%, respectively, compared with that of the HC treatment group. There was no significant difference in the apoptosis rate between the thiram group and the control group.

Compared with the control group, the G0/G1 phase of Mg63, Saos-2, K7M2-WT cells in the HC group increased by 15.2%, 8.8% and 4.8%, respectively, while the S phase decreased by 12.8%, 15.5% and 17.7%, respectively. Compared with the HC group, the average apoptosis rate in the drug combination group increased by 14.9%, 11.2%, and 21.3% and decreased by 15.4%, 13.3%, and 14.1% in S phase, respectively, as shown in Fig. 3B. Therefore, the combination of thiram and HC significantly promoted the cycle arrest of osteosarcoma cells compared with HC alone. There was no significant change in the cell cycle between the thiram group and the untreated group.

### Hydrocortisone treatment significantly altered the mRNA expression profile and Wnt/beta-catenin signaling pathways in osteosarcoma cells

**Fig. 4:**
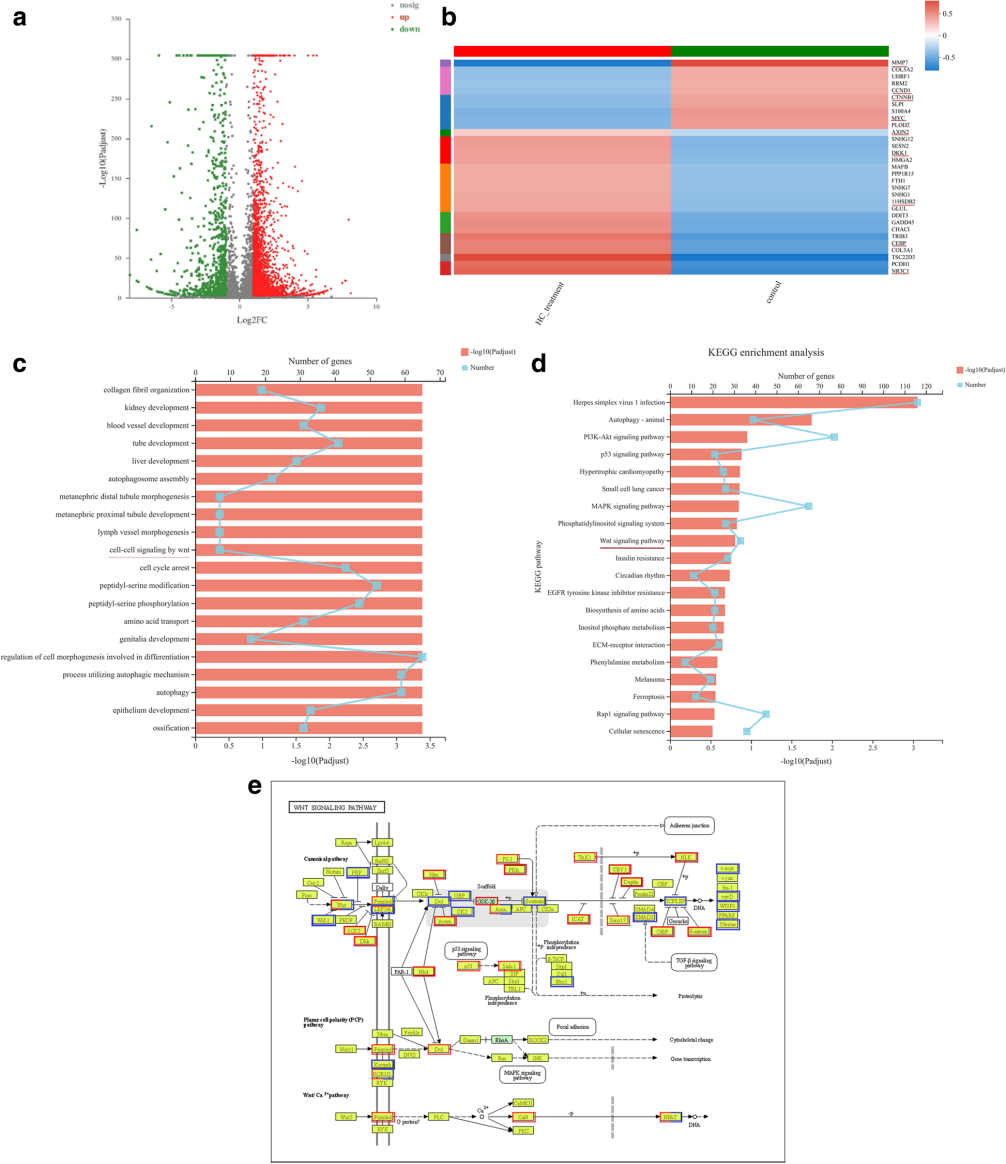
Changes in gene expression in different signaling pathways. **A** Volcano plot of gene expression differences. **B** Cluster heatmap of differential genes. **C** Functional enrichment analysis of differential genes in Gene Ontology. **D** The Kyoto Encyclopedia of Genes and Genomes (KEGG) Functional enrichment analysis of different genes. **E** Changes in gene transcription in the WNT signaling pathway

To explore the signal pathway changes caused by HC, we conducted transcriptome sequencing and bioinformatics analysis of untreated and HC-treated MG63 cells, as shown in Fig. 4. Cluster analysis of differentially expressed genes showed that HC significantly changed the mRNA expression profile of MG63 cells, as illustrated in Fig. 4A. Due to too many differentially expressed genes, we only selected genes related to Wnt/β-catenin signaling pathway for cluster heatmap analysis, and Fig. 4B was obtained. The results showed that 11HSD2, GCR (NR3C1), DKK1, and C/EBP were significantly upregulated, while beta-Catenin (CTNNB1), C-MYC (MYC), MMP7 and cyclin D1 (CCND1) were significantly downregulated. Bioinformatics analysis in Fig. 4 C-E show that the Wnt/β-catenin signaling pathway was significantly changed in HC-treated MG63 cells.

### Thiram enhances the inhibitory effects of HC through Wnt/β-catenin pathway

**Fig. 5:**
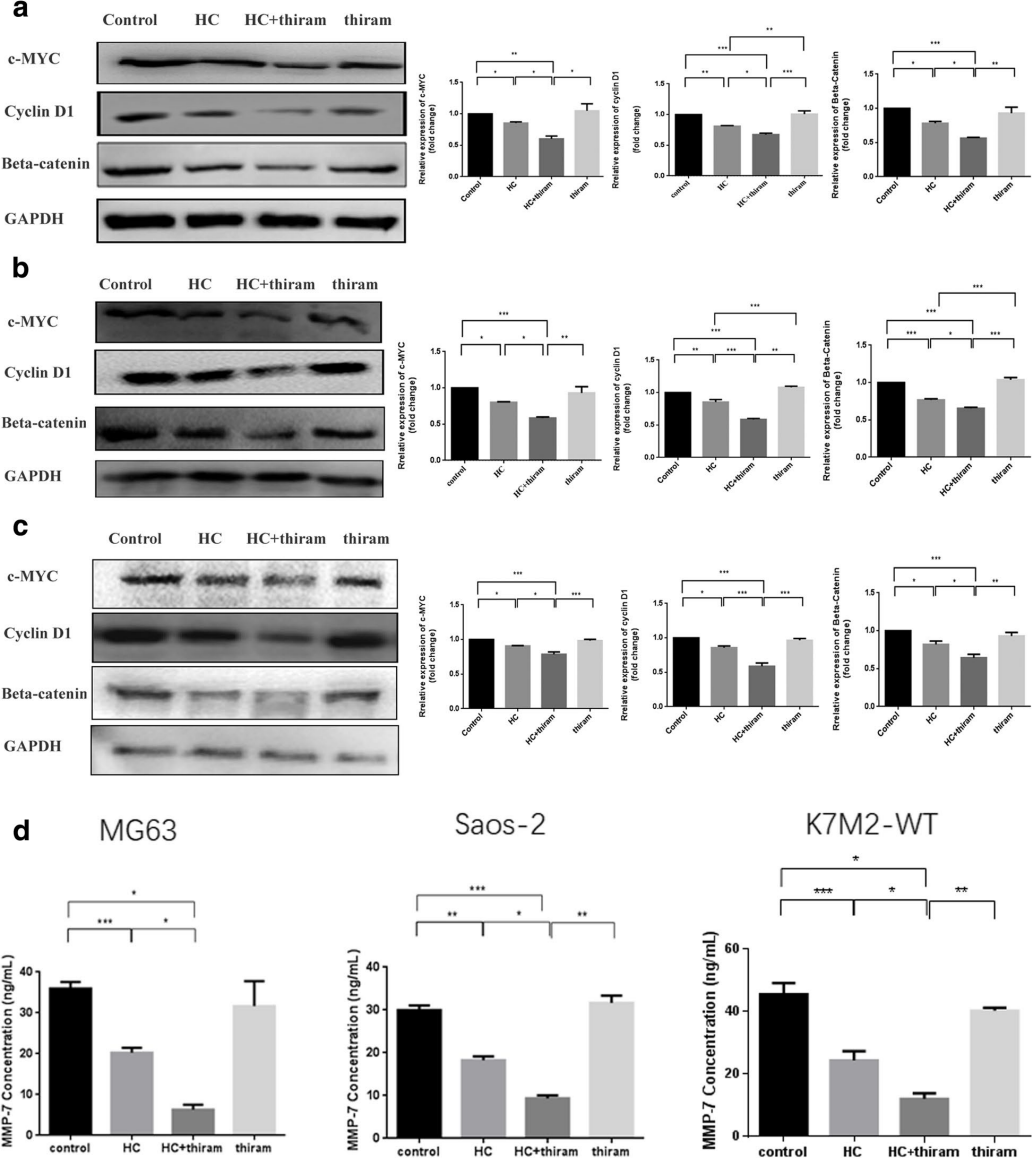
Effects of HC and thiram on proteins of Wnt/β-catenin pathway in osteosarcoma cells. **A** HC and thiram affect the proteins in Wnt/β-catenin pathway in Saos-2 cells. **B** HC and thiram affect the proteins in Wnt/β-catenin pathway in Mg63 cells. **C** HC and thiram affect the proteins in Wnt/β-catenin pathway of K7M2-WT cells. **D** Combined treatment with HC and thiram significantly reduced the MMP-7 concentration (left: MG63, middle: Saos-2, right: K7M2-wt). The histograms show the quantitative analysis of the samples. Measurement data were measured using the mean ± SD. The statistical significance of differences between two groups was analyzed using the unpaired t test. Repetition = 3 *p < 0.05, **p < 0.01, and ***p < 0.001

To verify the results of bioinformatics analysis, β-catenin (extracted from the cell nucleus), c-MYC, cyclin D1, MMP-7, proteins that were related to the apoptosis, proliferation, cell cycle and migration in the Wnt/β-catenin pathway were collected and detected by WB or ELISA as shown in Fig. 5A-D. The results showed that HC treatment significantly reduced the expression of c-MYC, Cyclin D1, beta-catenin and MMP-7, and these results are consistent with those of the bioinformatics analysis. After HC combined treatment with thiram, the protein expression of c-MYC, Cyclin-D1, β-catenin and MMP-7 was further decreased, and there were significant differences compared with the HC group. These results indicated that thiram combined with HC further inhibited osteosarcoma cells through the Wnt/beta-catenin pathway.

### Thiram inhibited 11HSD2 activity in osteosarcoma cells

**Fig. 6:**
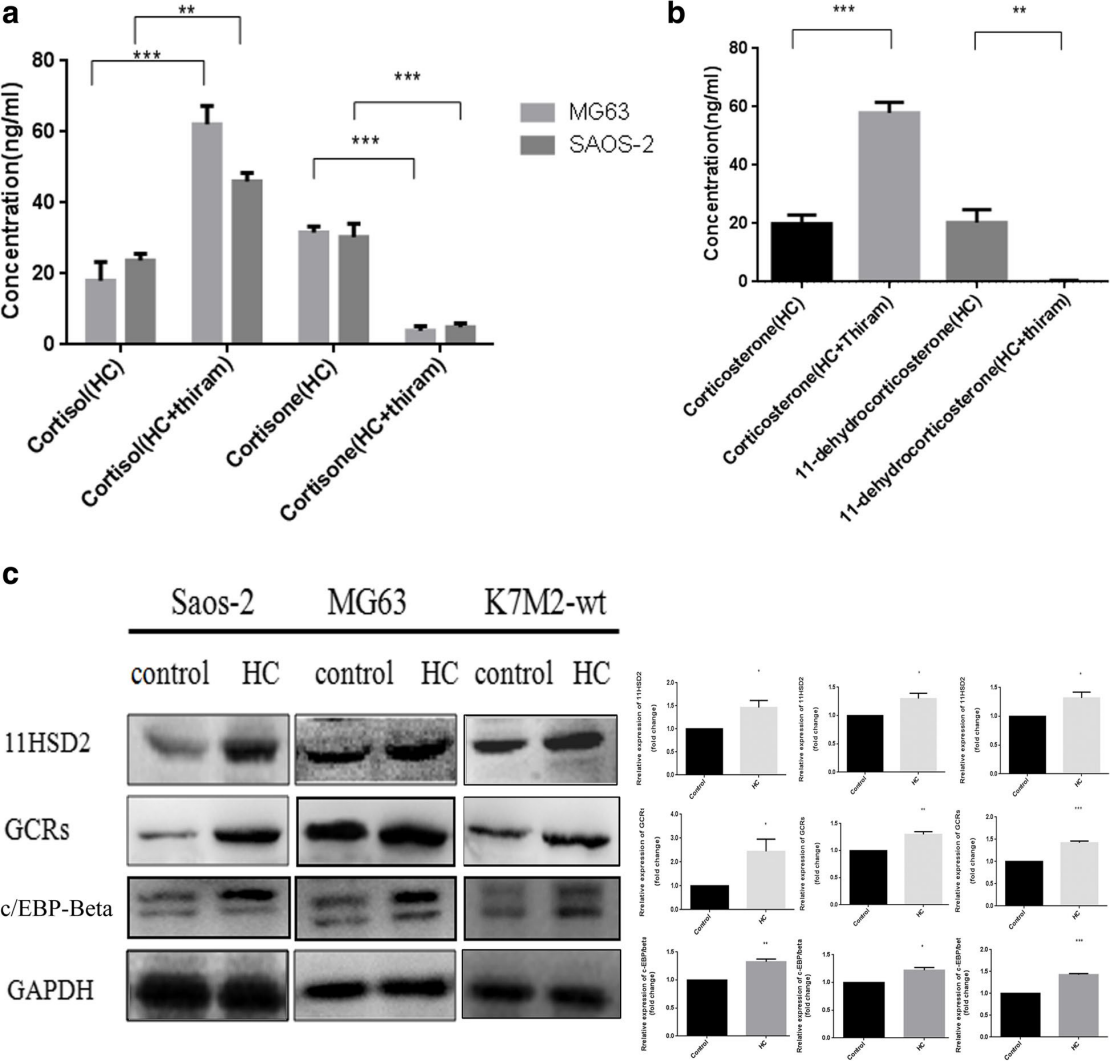
Effects of thiram on cells. **A** The levels of cortisol and cortisone in human osteosarcoma cells after treatment with HC. **B** The levels of corticosterone and 11-dehydrocorticosterone in mouse osteosarcoma cells after treatment with HC. **C** Dugs affected the expression of 11HSD2, GCR and c/EBP-beta. The histograms at the bottom are quantitative analyses of the samples. Measurement data were measured using the mean ± SD. The differences between two groups were analyzed using the unpaired t test. Repetition = 3 *p < 0.05, **p < 0.01. and ***p < 0.001

The results are shown in Fig. 6A, showing that the cortisol levels in MG63 and Saos-2 cells in the HC combined with thiram group were increased by 44.07 ng/ml and 22.29 ng/ml, and the cortisol levels were decreased by 27.75 ng/ml and 25.48 ng/ml, respectively, compared with those in the HC-treated group. Figure 6B shows corticosterone in K7M2-WT cells was elevated by 38.05 ng/ml and 11-dehydrocorticosterone was decreased by 20.15 ng/ml. These results indicated that thiram effectively inhibited 11HSD2 activity [25].

### The combination of HC and thiram promoted the expression of 11HSD2, GCR and c/EBP-beta

After the three osteosarcoma cell lines were treated with thiram and HC for 48 h, total proteins were collected for WB, and Fig. 6C was obtained. The results showed that the expression of GCR, C/EBP-Beta and 11HSD2 increased after HC treatment.

11HSD2 is a tissue-level glucocorticoid regulating enzyme. The increased expression of 11HSD2 induced by HC increases the metabolic capacity of the body to exogenous HC, which may be one of the reasons for the body's drug resistance to HC.

### The combination of si-11HSD2 and HC promoted the inhibitory effect of HC on osteosarcoma cells through Wnt/β-catenin pathway analysis

**Fig. 7:**
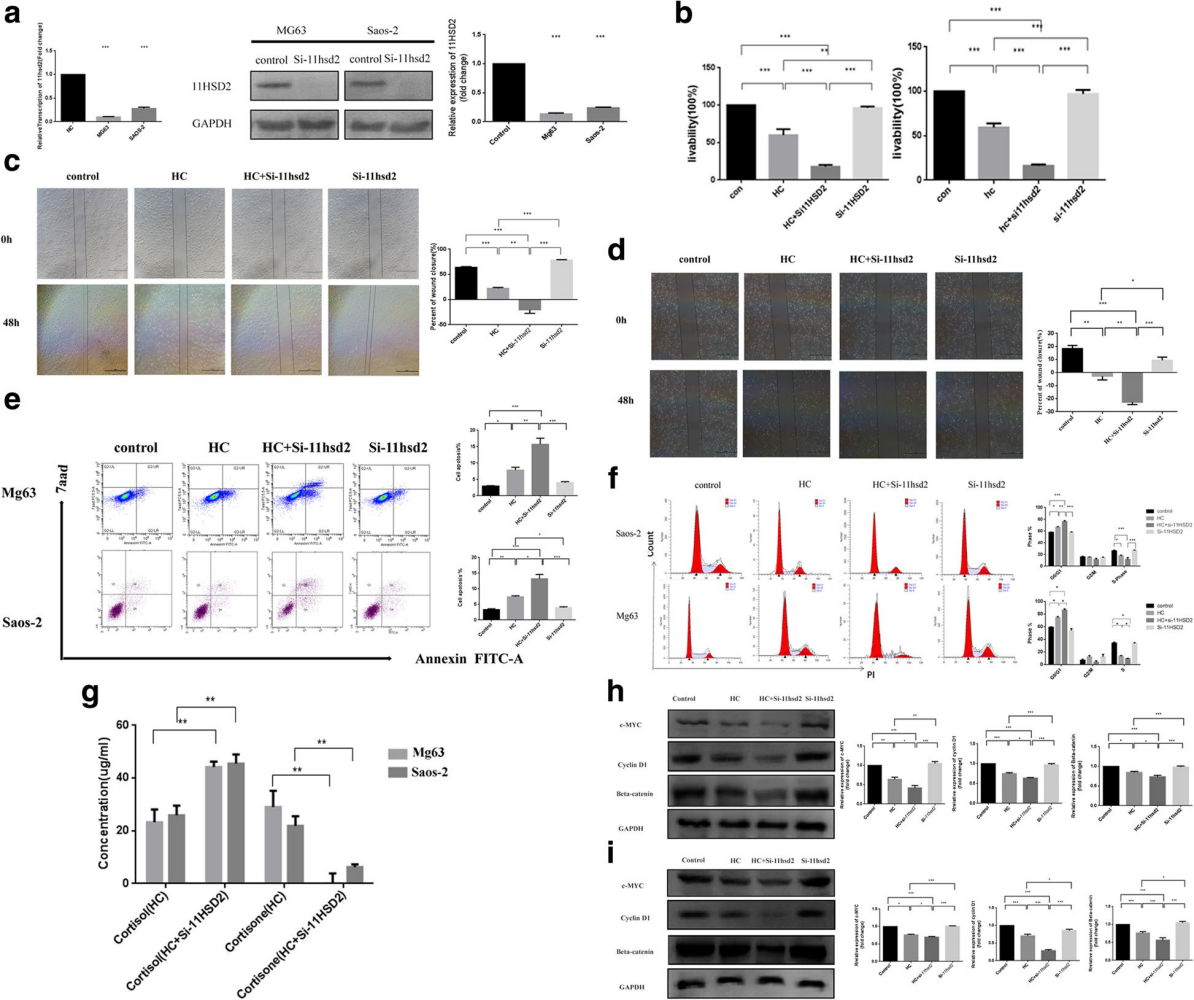
Effect of si-11HSD2 combined with HC on osteosarcoma cells. **A** The 11HSD2 expression level was determined by reverse transcription-quantitative PCR and WB in MG63 and Saos-2 cells. **B** Effect of drugs on the viability of human osteosarcoma cells: Left: Mg63, right: Saos-2. **C** Effect of drugs on the migration of Mg63 cells. **D** Effect of drugs on the migration of Saos-2 cells. **E** The combination of HC and siRNA increased apoptosis in osteosarcoma cells. **F** The combination of HC and siRNA increased cell arrest in osteosarcoma cells. **G** The levels of cortisol and cortisone in osteosarcoma cells after treatment with HC and/or si-11HSD2. **H** HC and/or siRNA affected the expression of proteins in Wnt/β-catenin pathway in Saos-2 cells. **I** HC and/or siRNA affect the proteins in Wnt/β-catenin pathway in Mg63 cells

SiRNA targeting the 11HSD2 gene was designed and transfected into MG63 and Saos-2 cells. The silencing effect of 11HSD2 was verified by Q-PCR, as shown in Fig. 7A, which showed that the transcription of the 11HSD2 gene was significantly reduced 48 h after transfection.

After si-11HSD2 silencing, OS cells were treated with HC. After 48 h, cell proliferation, migration, apoptosis and cell cycle changes were analyzed, and the data are shown in Fig. 7B-F. The results showed that the proliferation and migration in the si-11HSD2 combined with HC group were significantly decreased, and apoptosis and cell cycle inhibition were significantly increased compared with those in the HC group alone.

After transfection of si-11HSD2, cells were treated with HC for 48 h, and the concentrations of cortisone and cortisol before and after treatment were detected by ELISA, as shown in Fig. 7G. The results showed cortisol in the silenced group was significantly higher than that in the nonsilenced group, while corticosterone was significantly lower. This suggests that silencing 11HSD2 can significantly increase cortisol in cells, which is consistent with the effect of thiram.

Figure 7H and I show that the expression of c-MYC, beta-Catenin and cyclin D1 decreased after 11HSD2 silencing by siRNA and HC treatment. Thus, the proliferation, apoptosis and cell cycle of osteosarcoma cells were further inhibited compared with HC alone.

## Discussion

This is the first preclinical study to report that HC can inhibit osteosarcoma and that thiram can enhance HC's inhibition of osteosarcoma. This effect is due to the protein expression changes in Wnt/β-catenin pathway after HC treatment and thiram inhibition of 11HSD2.

After HC treatment, the proliferation and migration of osteosarcoma cells were inhibited, and the volume of osteosarcoma in mice was significantly smaller than that in the untreated group. Flow cytometry showed that HC could induce apoptosis and cycle arrest in osteosarcoma cells. Transcriptional sequencing, WB and ELISA results showed that the expression of β-catenin, MMP7, c-MYC and cyclin-D1 in HC-treated cells was lower than that in the untreated group. The molecular mechanism may be that HC, as a glucocorticoid drug, can bind to the glucocorticoid response element (GRE) sequence of the DKK-1 promoter to enhance the transcription of DKK-1 [26]. DKK-1 negatively regulates β-catenin by binding to cell surface receptors such as LRP5/6 and Kremen1/2 [27]. β-catenin, a key protein of the Wnt/β-catenin signaling pathway [28], can enter the nucleus and bind to lymphoid enhancer factor/T-cell factor (LEF/TCF) [29], a lymphoid enhancer factor/T-cell factor LEF/TCF ([30]), and activate the expression of the downstream target genes MMP7 [31], c-MYC [32] and Cyclin-D1 [33]. Therefore, HC inhibits tumor cells by inhibiting the Wnt/β-catenin signaling pathway.

In clinical applications, patients with synthetic glucocorticoids such as HC and dexamethasone are prone to develop drug resistance after long-term use [34], which reduces the therapeutic effect of drugs [35]. Therefore, we further discuss how to reduce glucocorticoid resistance. Bioinformatics analysis and WB results showed that the expression of GCR, C/EBP-Beta and 11HSD2 was significantly increased in HC-treated osteosarcoma cells, resulting in HC resistance. The reasons may be that HC acts on the GRE recognition region of GCR to induce increased expression of GCR [36–38], and GCR upregulates C/EBP-beta by interacting with C/EBP-beta in the nucleus [39–42]. C/EBP-Beta acts as a transcription factor to bind to the 11HSD2 promoter, increasing the expression of 11HSD2 [41], thus increasing the metabolic capacity of cells to HC and forming a drug-resistant loop. Therefore, reducing the activity of 11HSD2 may reduce HC resistance and improve HC efficacy.

Thiram is an artificial compound with low cost, a simple production process and a stable chemical structure. It can specifically inhibit the enzyme activity of 11HSD2 in humans and is an ideal 11HSD2 inhibitor [43]. In recent years, it has been reported that it can be used to inhibit tumors [44, 45].

We treated osteosarcoma cells with thiram and HC, and the results showed that thiram effectively reduced the production of cortisone and 11-dehydrocorticosterone and increased cortisol and corticosterone in osteosarcoma cells, that is, thiram effectively inhibited the activity of 11HSD2.

Subsequent studies showed that the combination of thiram and HC further inhibited the proliferation and migration of osteosarcoma cells, increased apoptosis and cycle arrest, and further decreased the expression of beta-catenin, c-MYC, Cyclin-D1 and MMP7 compared with HC alone.

These results suggest that the 11HSD2 activity of osteosarcoma cells was inhibited and that the glucocorticoid concentration increased after HC and thiram treatment, which better inhibited OS cells from the Wnt/β-catenin pathway.

After silencing 11HSD2 with small interfering RNA, osteosarcoma cells were treated with HC. The results were consistent with the experimental results of HC combined with thiram, suggesting that inhibition of 11HSD2 is important for HC curing of osteosarcoma. These results are consistent with reports that 11HSD2 is highly expressed in osteosarcoma cells and human osteosarcoma tissues [46] and that the activity of 11HSD1 could not be detected in human osteosarcoma tissues, but only 11HSD2 and the expression of 11HSD2 were significantly correlated with poor prognosis of chemotherapy [47].

In conclusion, this study found that HC inhibits the proliferation and migration of osteosarcoma cells through the Wnt/β-catenin pathway and induces apoptosis and cycle arrest. However, HC can initiate the GCR/C/EBP-Beta/11HSD2 loop and increase the expression of 11HSD2, resulting in drug resistance. As a synergist, thiram can inhibit 11HSD2 activity, reduce HC resistance and further inhibit osteosarcoma cells from the Wnt/β-catenin pathway.

To date, the inhibitory effect of HC on osteosarcoma has not been reported, nor have GC metabolic enzyme inhibitors been used to treat osteosarcoma. This study first proposed and confirmed that the activation of glucocorticoid metabolic enzymes may be a major mechanism of hydrocortisone resistance, and glucocorticoid metabolic enzyme inhibitors combined with glucocorticoids may be an efficient means in the treatment of osteosarcoma, providing new ideas for the reduction of glucocorticoid resistance in osteosarcoma and other diseases.

## Conclusion

Our results suggest that hydrocortisone inhibits osteosarcoma through the Wnt/β-catenin pathway and that thiram promotes HC inhibition of osteosarcoma from the same pathway by inhibiting 11HSD2 activity. This study provides new ideas for thiram to reduce hydrocortisone resistance and to treat osteosarcoma.

## Supplementary Information

**Additional file 1.**

**Additional file 2.**

**Additional file 3.**

**Additional file 4.**

**Additional file 5.**

**Additional file 6.**

**Additional file 7.**

## Data Availability

The datasets generated and/or analyzed during the current study are available in the ArrayExpress repository, E-MTAB-12398 or the reviewer’s link (https://www.ebi.ac.uk/biostudies/arrayexpress/studies/E-MTAB-12398?key=8ce6c006-d504-4faa-9928-f176b14976ee).
