# Supplementary figures and images for: Thiram, an inhibitor of 11ß-hydroxysteroid dehydrogenase type 2, enhances the inhibitory effects of hydrocortisone in the treatment of osteosarcoma through Wnt/β-catenin pathway
Source: BMC Pharmacol Toxicol. 2023 Mar 28;24:20. doi: 10.1186/s40360-023-00655-0 (PMC10045229; doi:10.1186/s40360-023-00655-0)

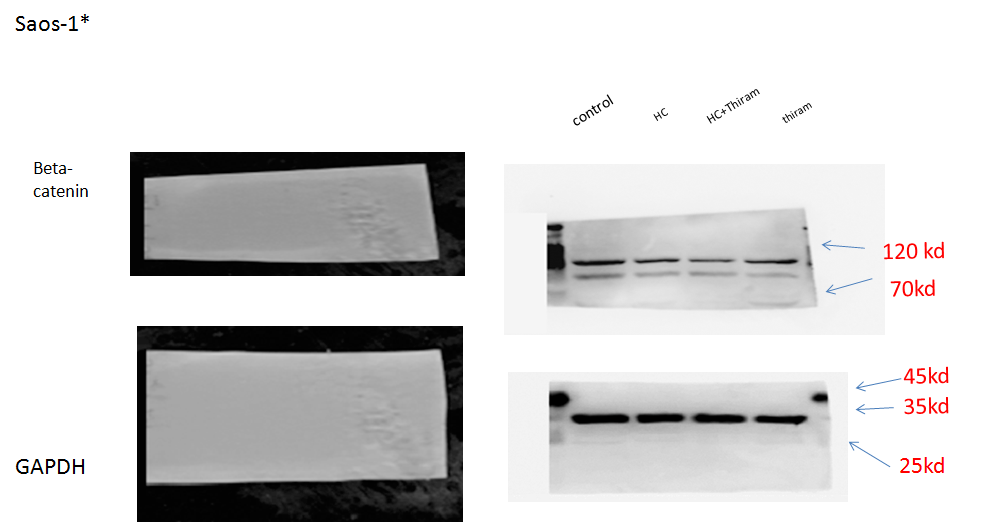

Supplement: Supplementary file 1 — Additional file 1. [file 40360_2023_655_MOESM1_ESM.zip › fig5a original/beta-catenin/1.tif]

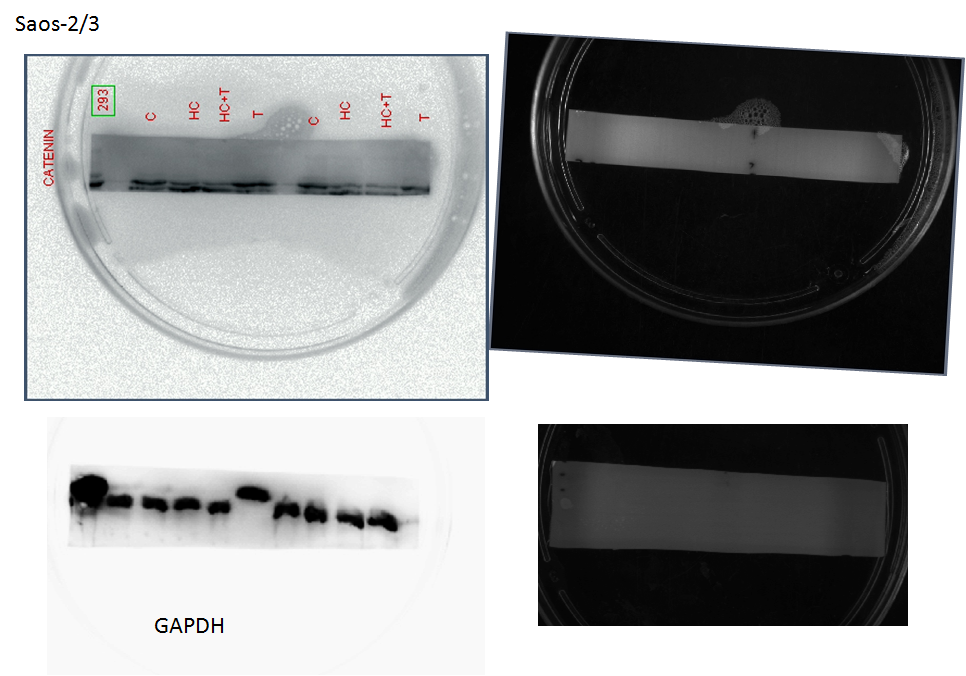

Supplement: Supplementary file 1 — Additional file 1. [file 40360_2023_655_MOESM1_ESM.zip › fig5a original/beta-catenin/2&3.tif]

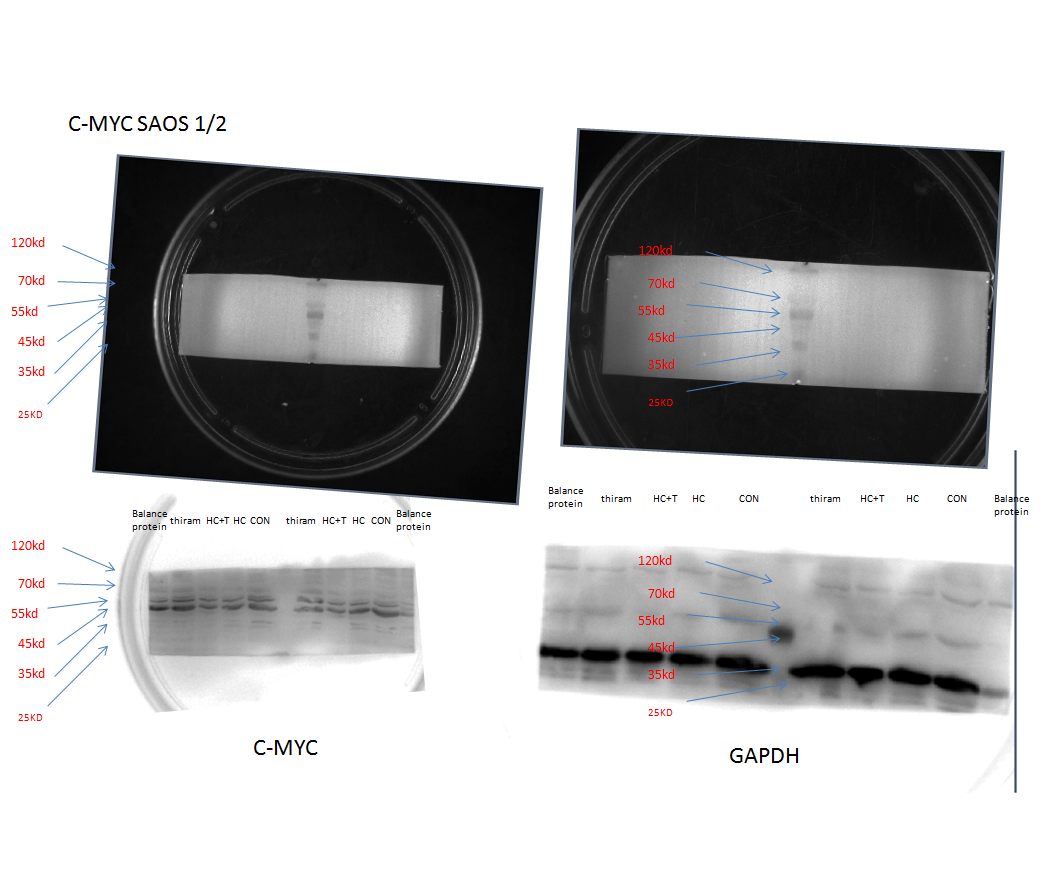

Supplement: Supplementary file 1 — Additional file 1. [file 40360_2023_655_MOESM1_ESM.zip › fig5a original/cmyc/1&2.tif]

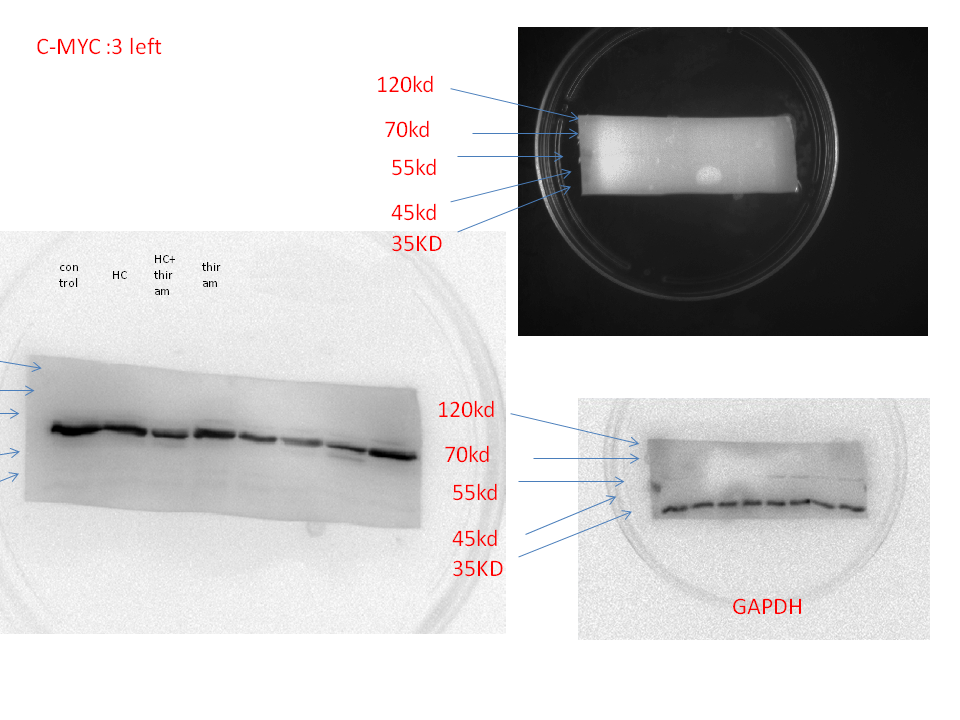

Supplement: Supplementary file 1 — Additional file 1. [file 40360_2023_655_MOESM1_ESM.zip › fig5a original/cmyc/3.tif]

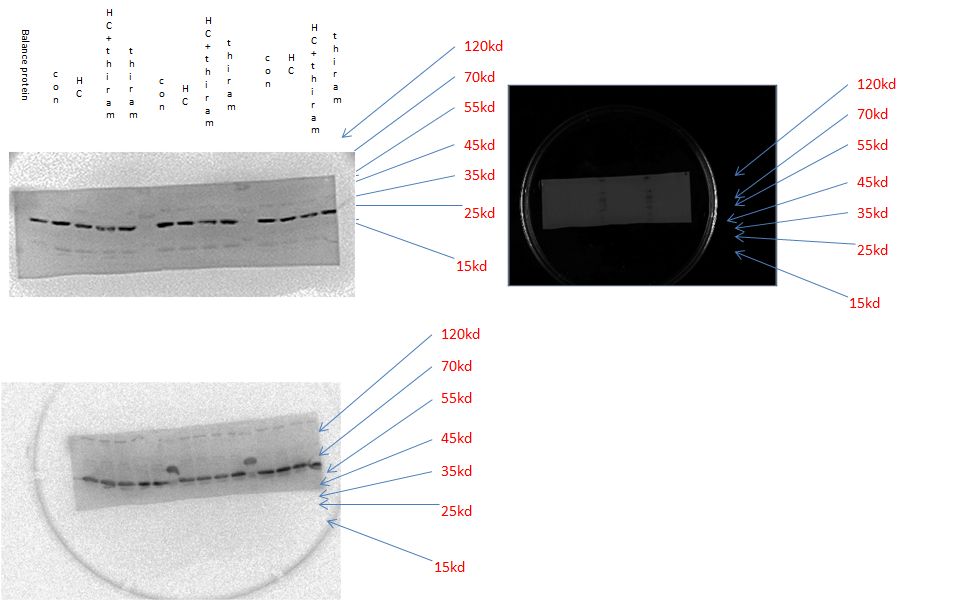

Supplement: Supplementary file 1 — Additional file 1. [file 40360_2023_655_MOESM1_ESM.zip › fig5a original/cyclinD1/saos2.tif]

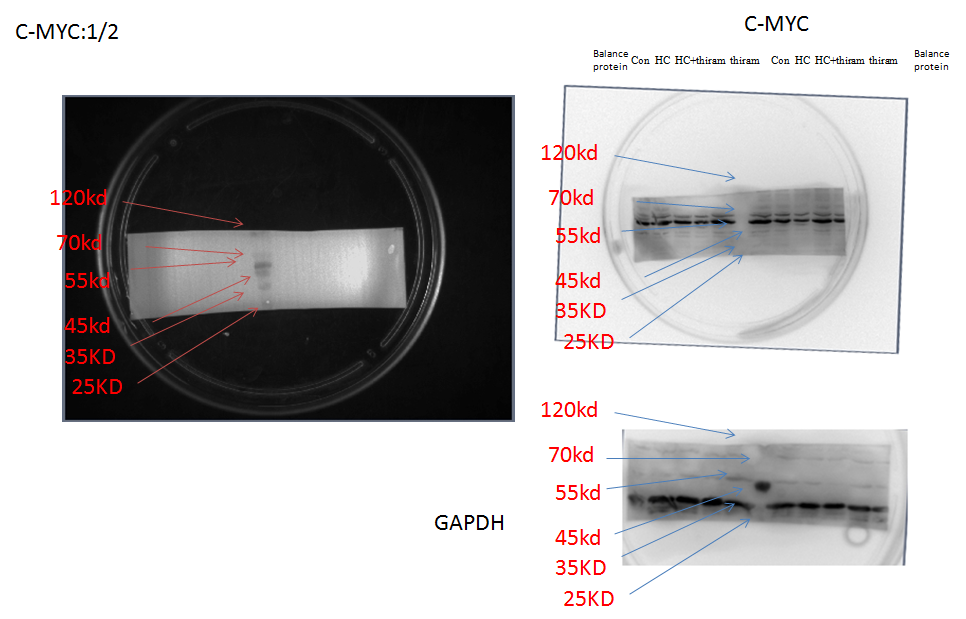

Supplement: Supplementary file 1 — Additional file 1. [file 40360_2023_655_MOESM1_ESM.zip › fig5b origin/CMYC/1.tif]

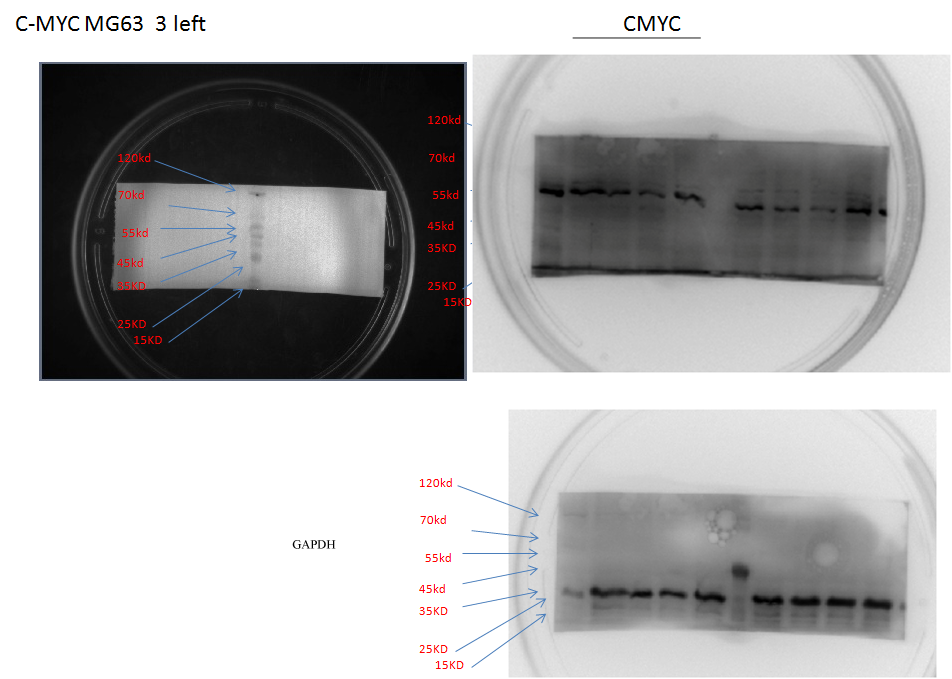

Supplement: Supplementary file 1 — Additional file 1. [file 40360_2023_655_MOESM1_ESM.zip › fig5b origin/CMYC/2.tif]

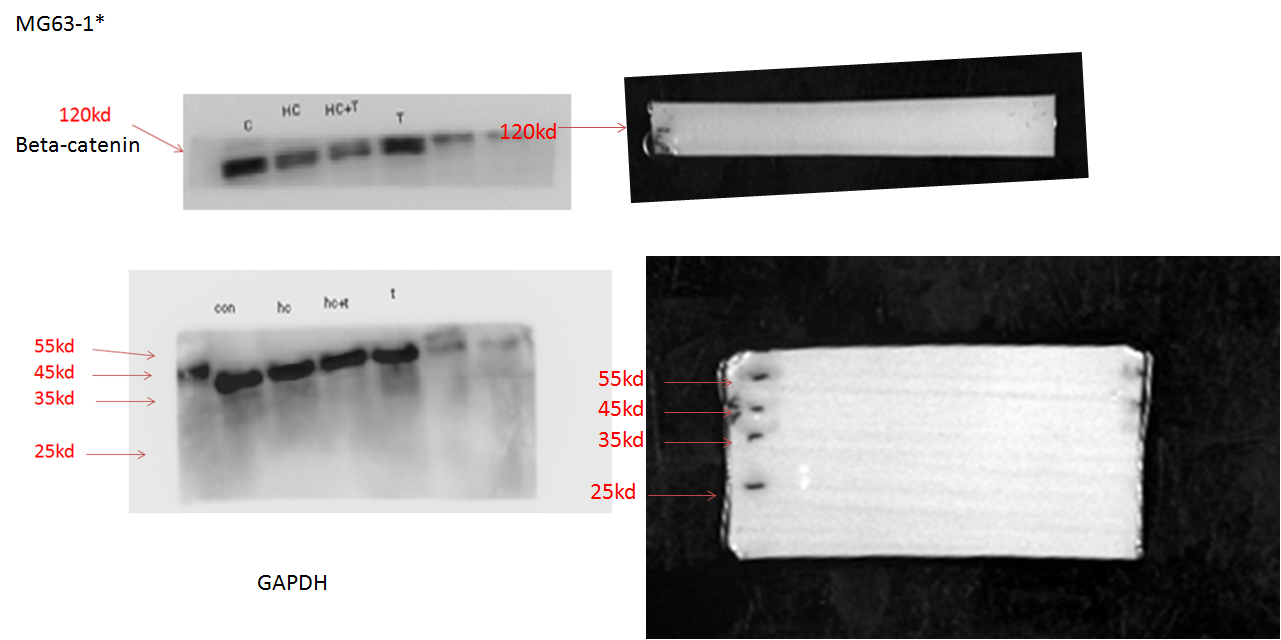

Supplement: Supplementary file 1 — Additional file 1. [file 40360_2023_655_MOESM1_ESM.zip › fig5b origin/beta-catenin/1.tif]

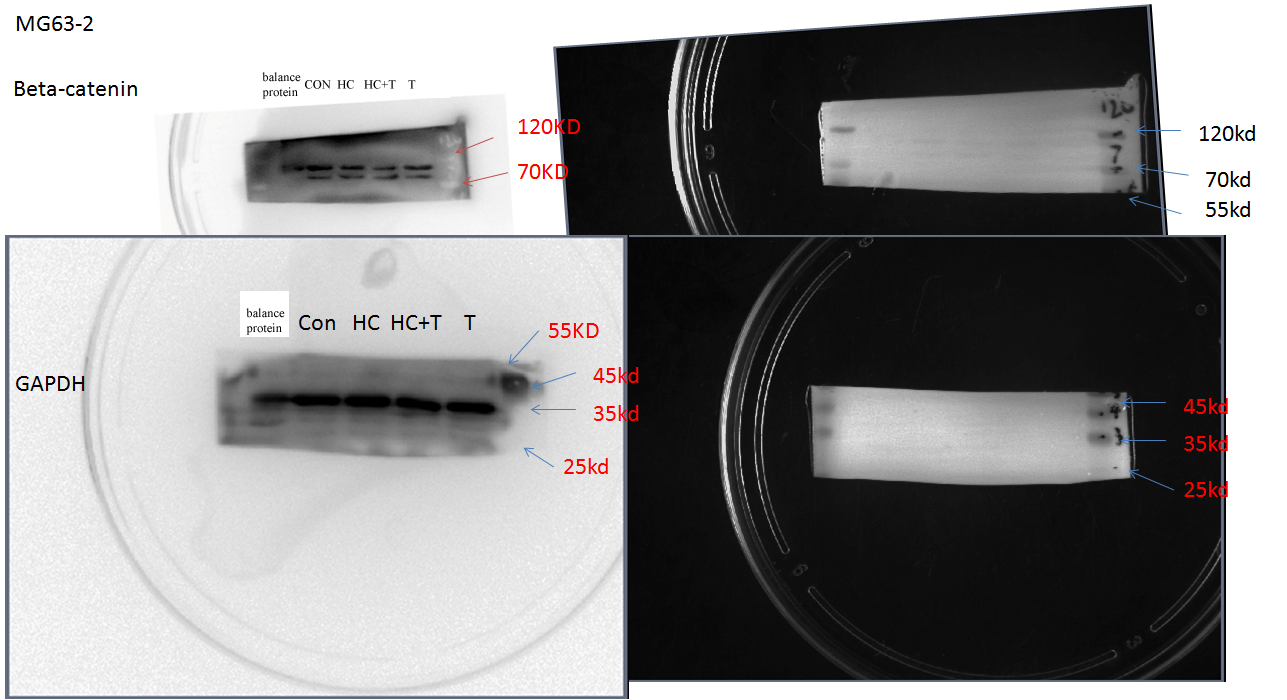

Supplement: Supplementary file 1 — Additional file 1. [file 40360_2023_655_MOESM1_ESM.zip › fig5b origin/beta-catenin/2.tif]

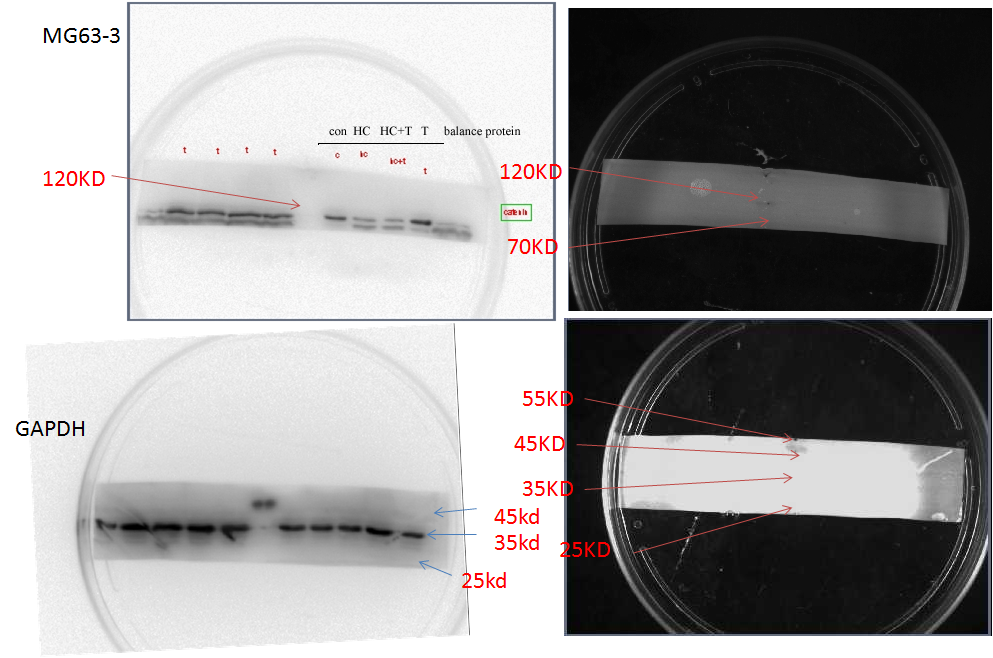

Supplement: Supplementary file 1 — Additional file 1. [file 40360_2023_655_MOESM1_ESM.zip › fig5b origin/beta-catenin/3.tif]

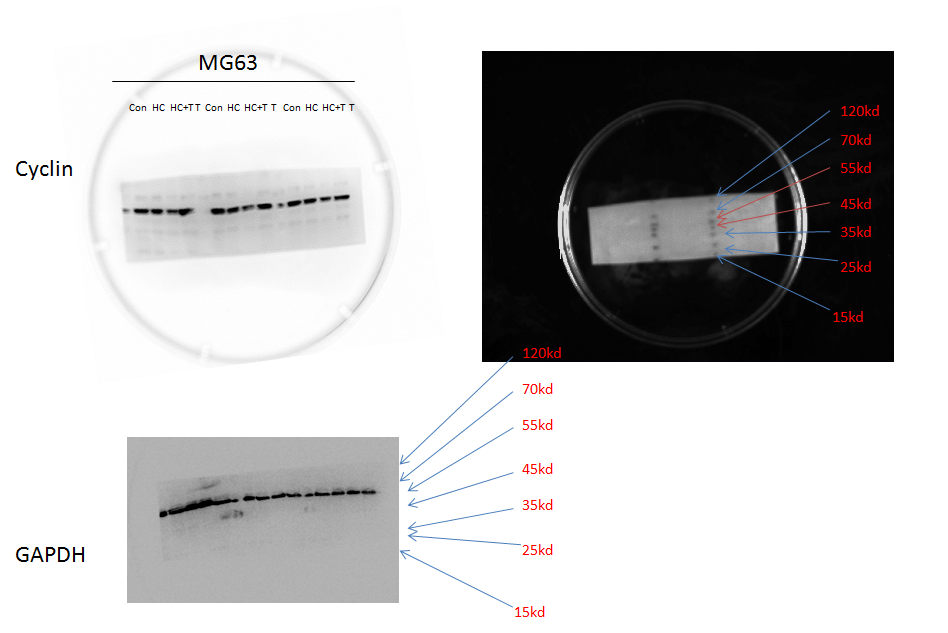

Supplement: Supplementary file 1 — Additional file 1. [file 40360_2023_655_MOESM1_ESM.zip › fig5b origin/cyclinD1/Cyclin.tif]

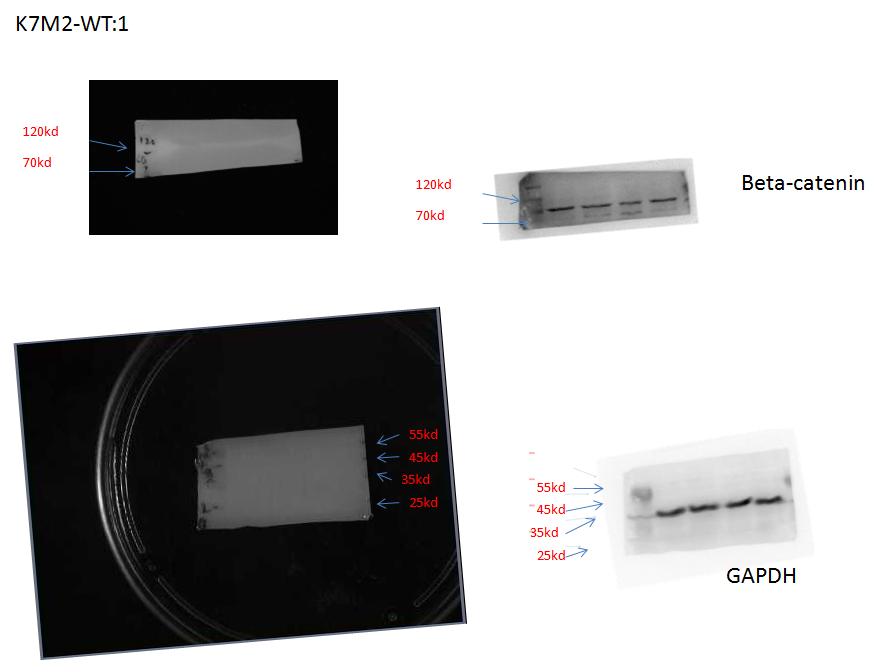

Supplement: Supplementary file 1 — Additional file 1. [file 40360_2023_655_MOESM1_ESM.zip › fig5c origin/beta-catenin/1.tif]

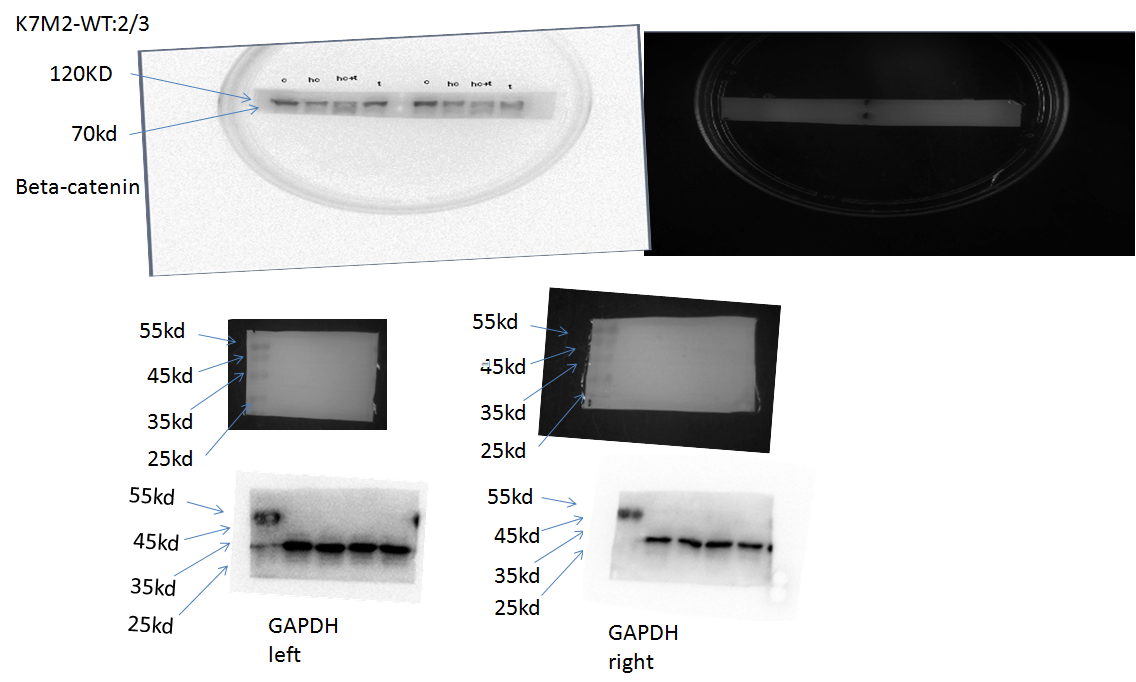

Supplement: Supplementary file 1 — Additional file 1. [file 40360_2023_655_MOESM1_ESM.zip › fig5c origin/beta-catenin/2.tif]

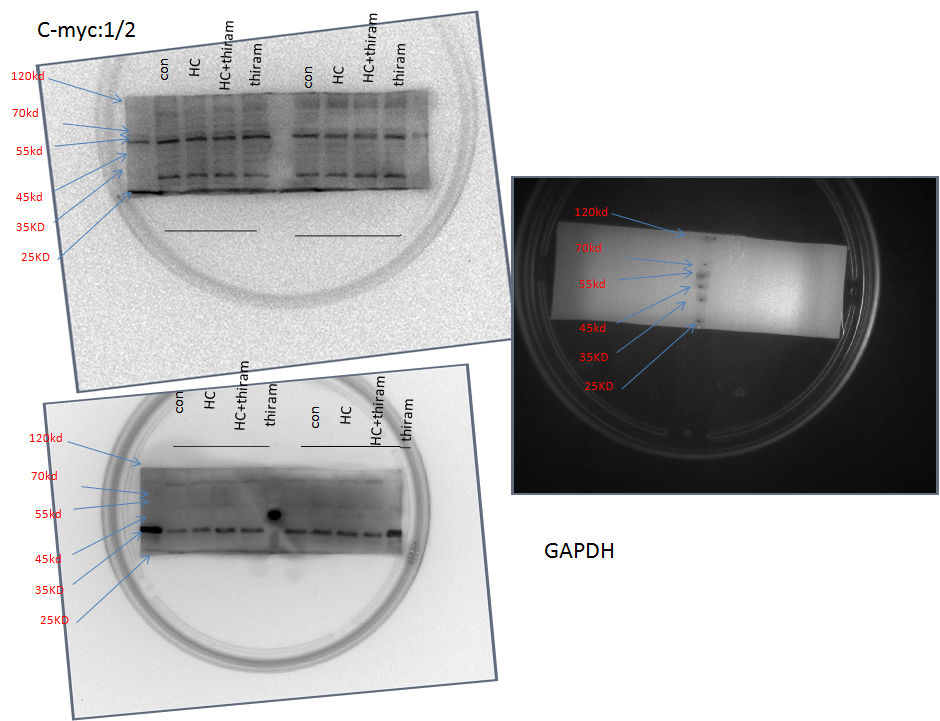

Supplement: Supplementary file 1 — Additional file 1. [file 40360_2023_655_MOESM1_ESM.zip › fig5c origin/cmyc/k7m2 1&2.tif]

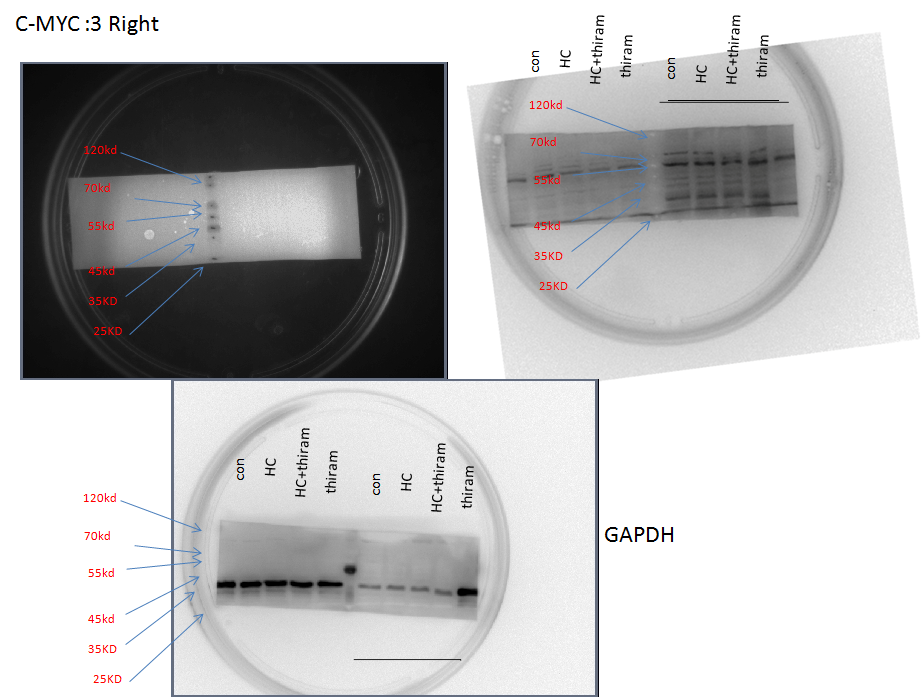

Supplement: Supplementary file 1 — Additional file 1. [file 40360_2023_655_MOESM1_ESM.zip › fig5c origin/cmyc/k7m2 3.tif]

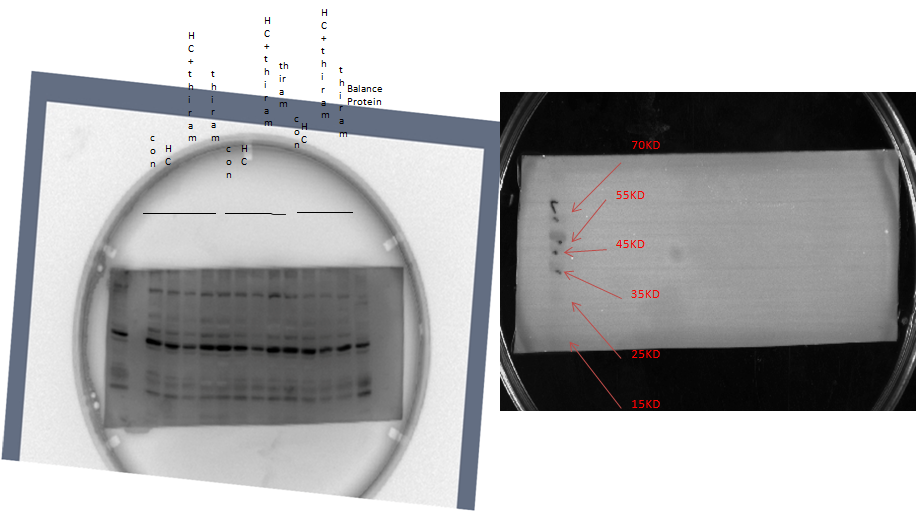

Supplement: Supplementary file 1 — Additional file 1. [file 40360_2023_655_MOESM1_ESM.zip › fig5c origin/cyclin d1/123.tif]

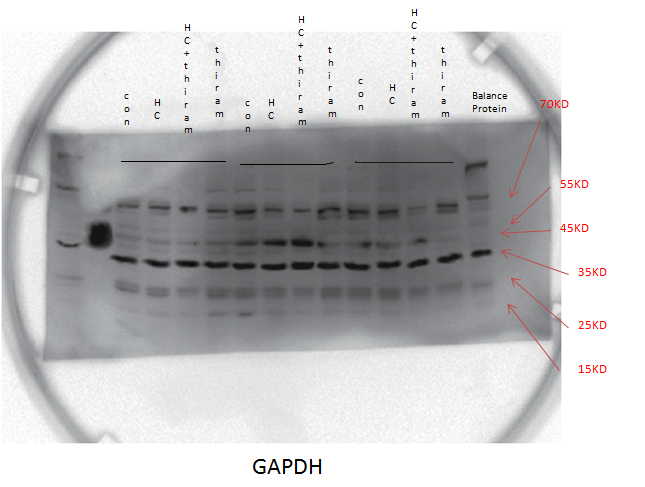

Supplement: Supplementary file 1 — Additional file 1. [file 40360_2023_655_MOESM1_ESM.zip › fig5c origin/cyclin d1/GAPDH.tif]

**1. 11HSD2**


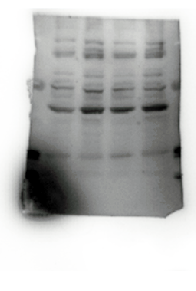

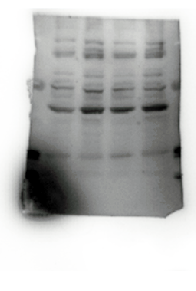


K7M2-wt

Saos-2


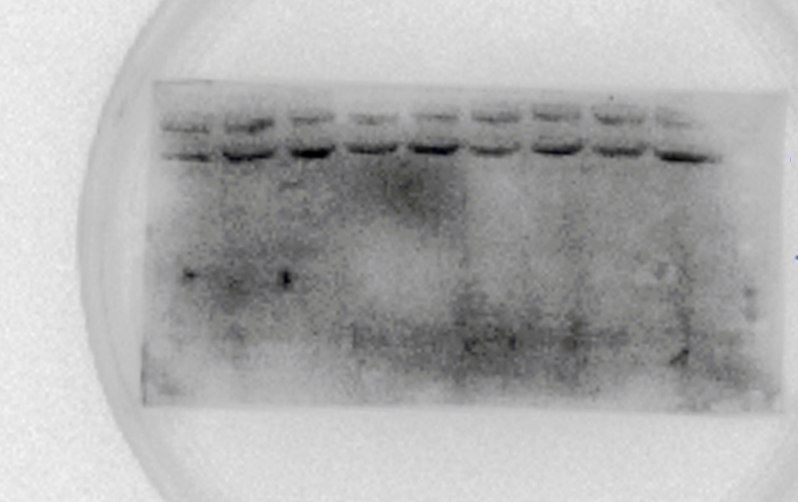


Mg63

**2、GCRs:**

**
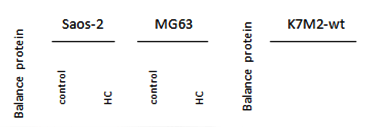
**


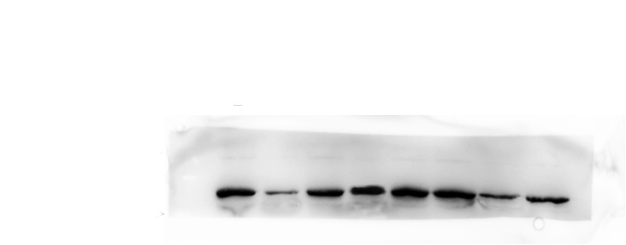


**3、c-EBP:**

**
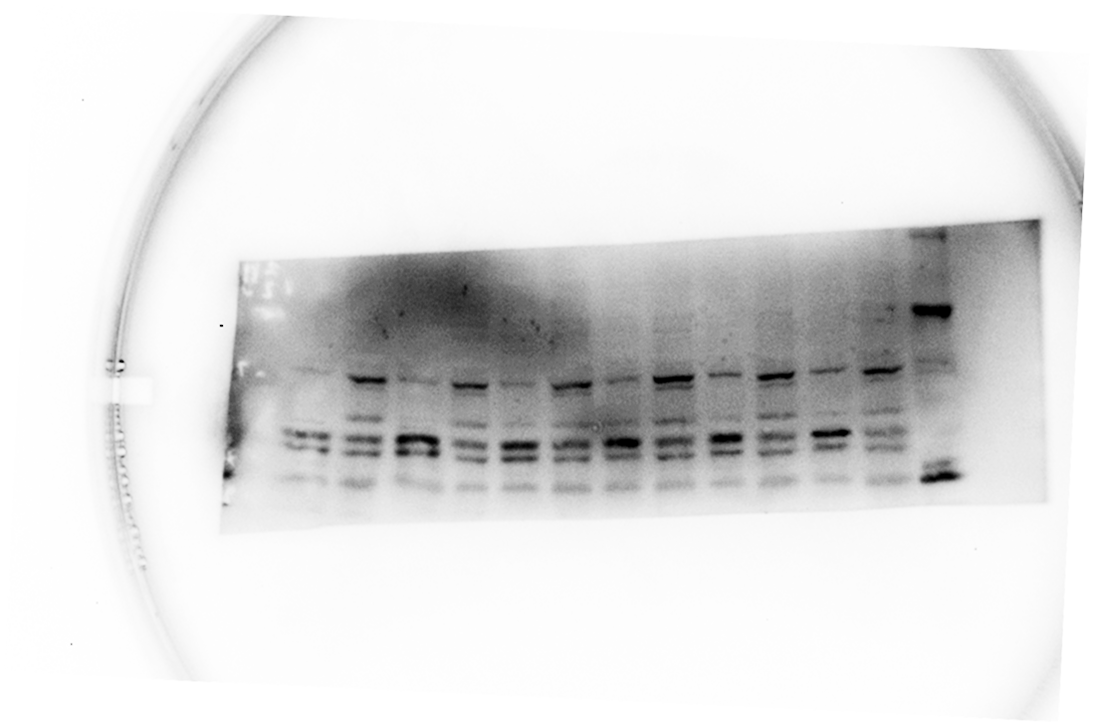
**

**Saos2 Mg63**

**
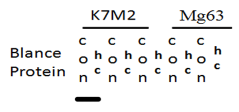
**

**
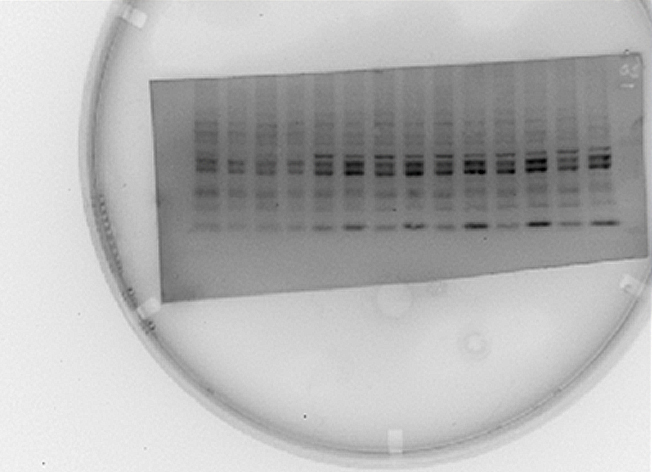
**

**3、GAPDH**

**
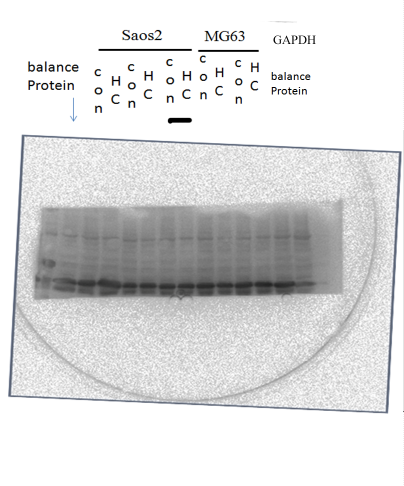
**

**Saos-2**

**
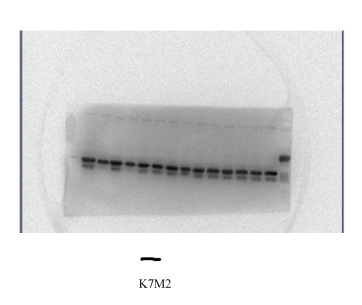
**

**
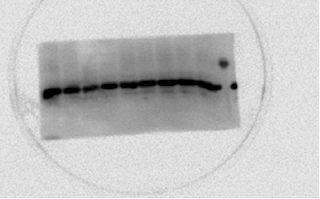
**

Mg63

Supplement: Supplementary file 5 — Additional file 5. [file 40360_2023_655_MOESM5_ESM.docx]

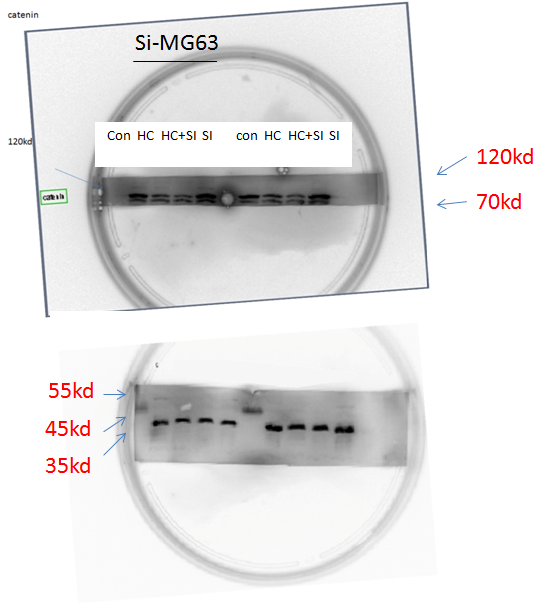

Supplement: Supplementary file 7 — Additional file 7. [file 40360_2023_655_MOESM7_ESM.zip › fig 7original/4group╠ß╜╗/MG63╠ß╜╗/beta-catenin╠ß╜╗/1.tif]

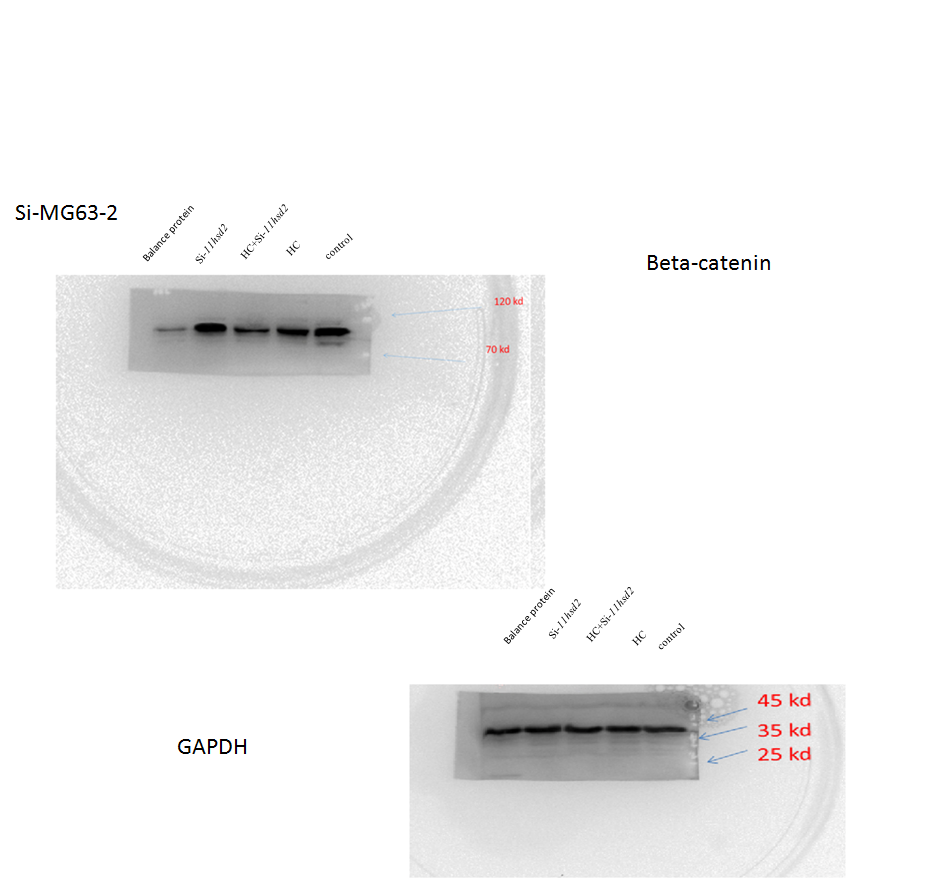

Supplement: Supplementary file 7 — Additional file 7. [file 40360_2023_655_MOESM7_ESM.zip › fig 7original/4group╠ß╜╗/MG63╠ß╜╗/beta-catenin╠ß╜╗/2.tif]

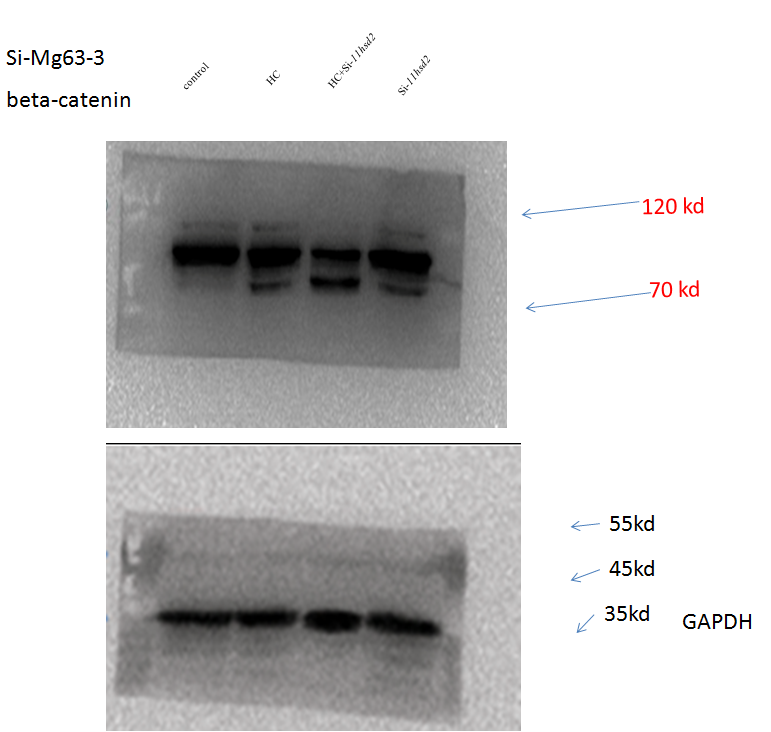

Supplement: Supplementary file 7 — Additional file 7. [file 40360_2023_655_MOESM7_ESM.zip › fig 7original/4group╠ß╜╗/MG63╠ß╜╗/beta-catenin╠ß╜╗/3.tif]

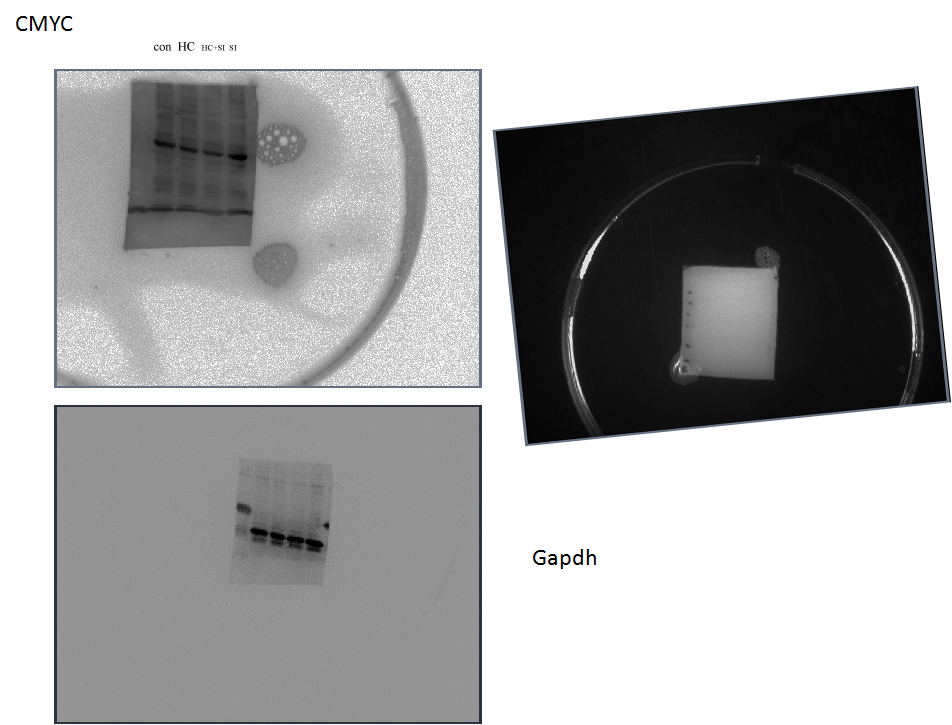

Supplement: Supplementary file 7 — Additional file 7. [file 40360_2023_655_MOESM7_ESM.zip › fig 7original/4group╠ß╜╗/MG63╠ß╜╗/cmyc╠ß╜╗/1.tif]

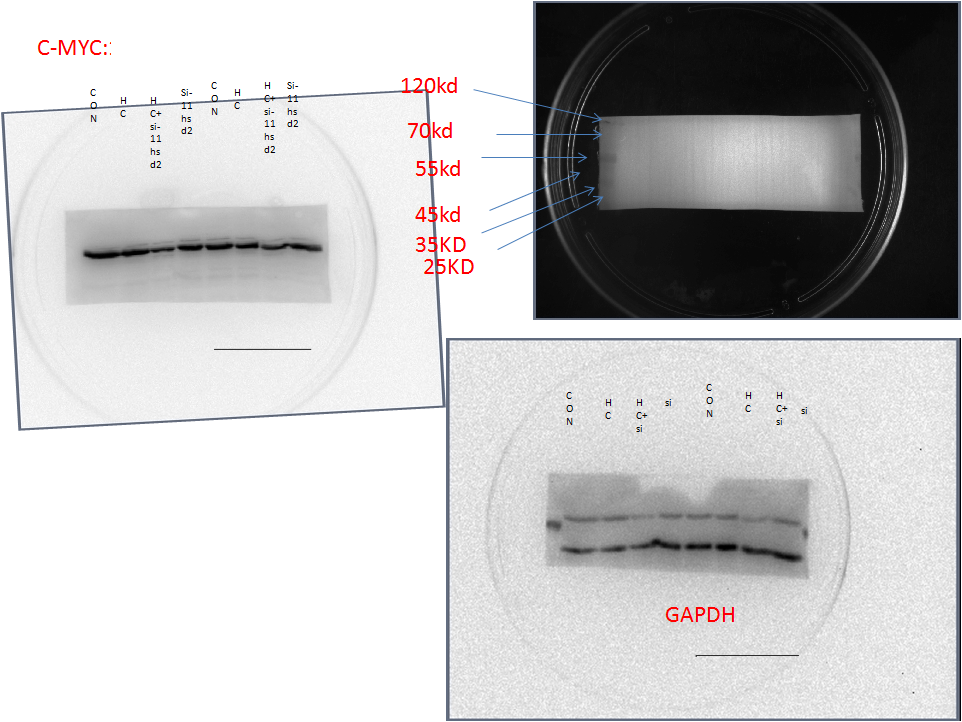

Supplement: Supplementary file 7 — Additional file 7. [file 40360_2023_655_MOESM7_ESM.zip › fig 7original/4group╠ß╜╗/MG63╠ß╜╗/cmyc╠ß╜╗/2.tif]

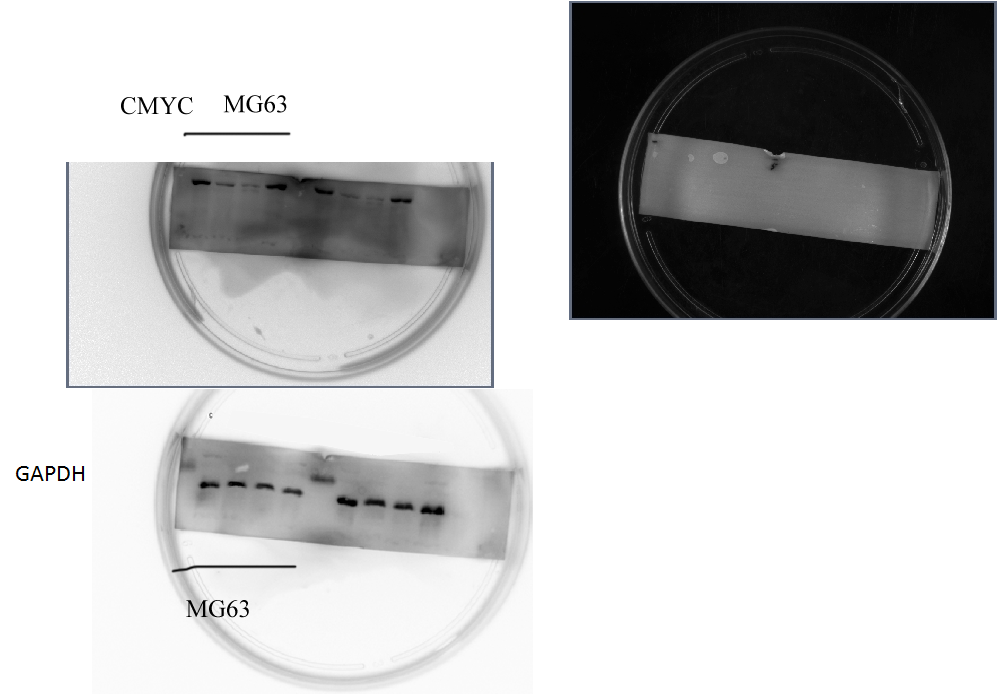

Supplement: Supplementary file 7 — Additional file 7. [file 40360_2023_655_MOESM7_ESM.zip › fig 7original/4group╠ß╜╗/MG63╠ß╜╗/cmyc╠ß╜╗/3.tif]

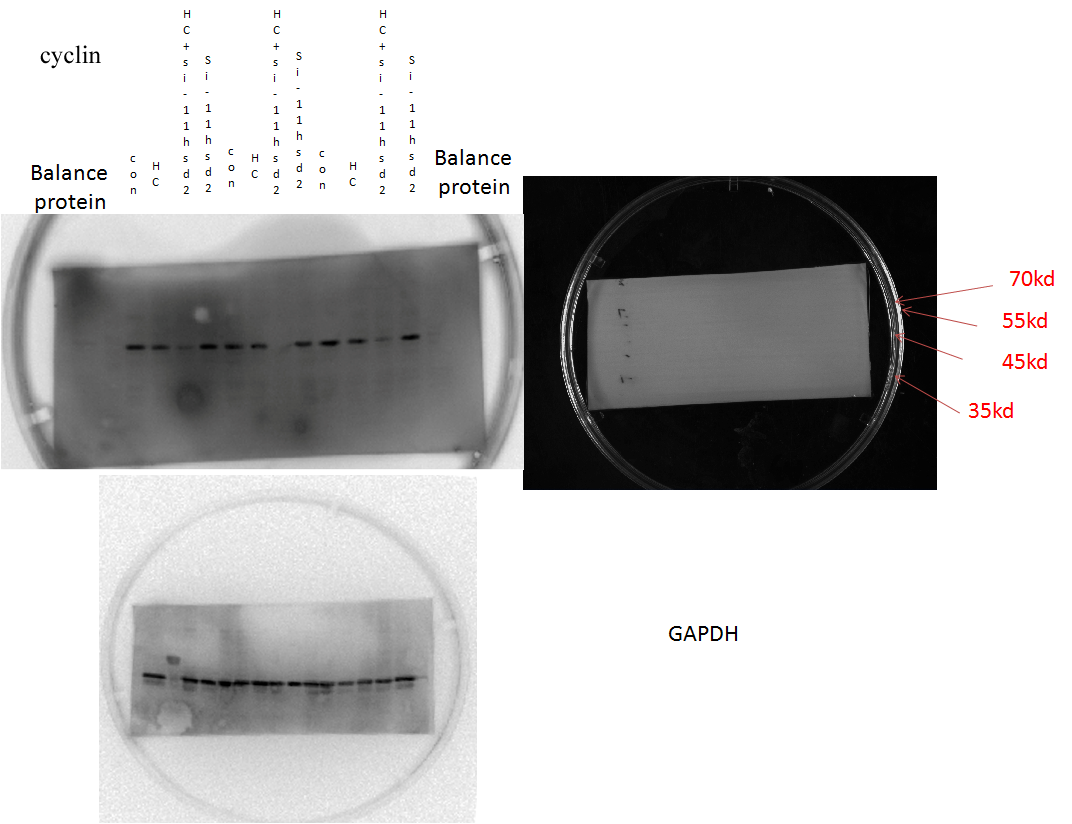

Supplement: Supplementary file 7 — Additional file 7. [file 40360_2023_655_MOESM7_ESM.zip › fig 7original/4group╠ß╜╗/MG63╠ß╜╗/cyclinD1╠ß╜╗/1.tif]

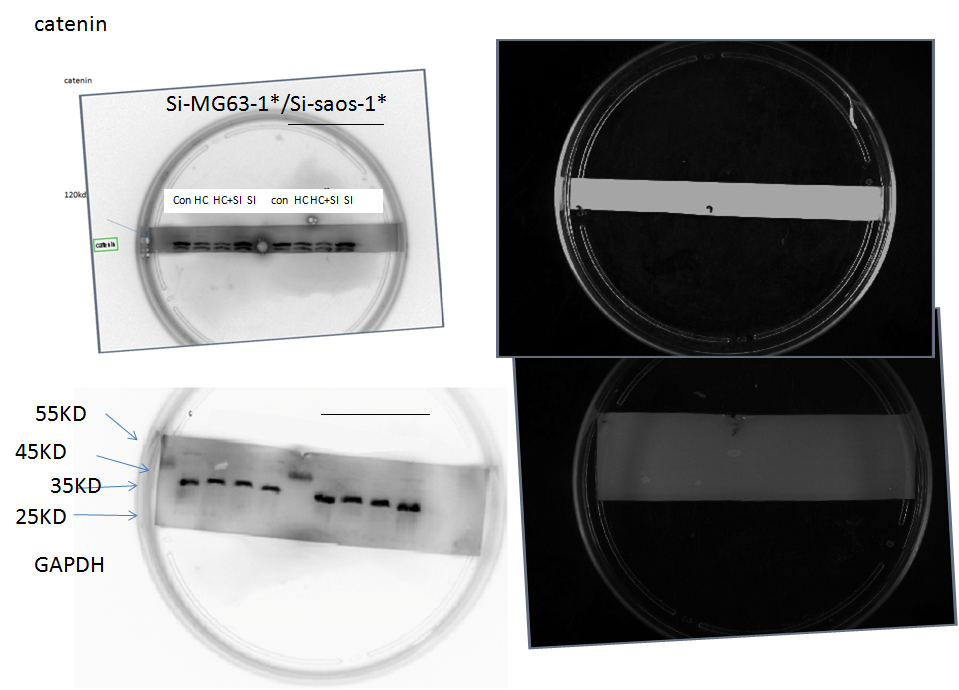

Supplement: Supplementary file 7 — Additional file 7. [file 40360_2023_655_MOESM7_ESM.zip › fig 7original/4group╠ß╜╗/Saos╠ß╜╗/beta-catenin/1.tif]

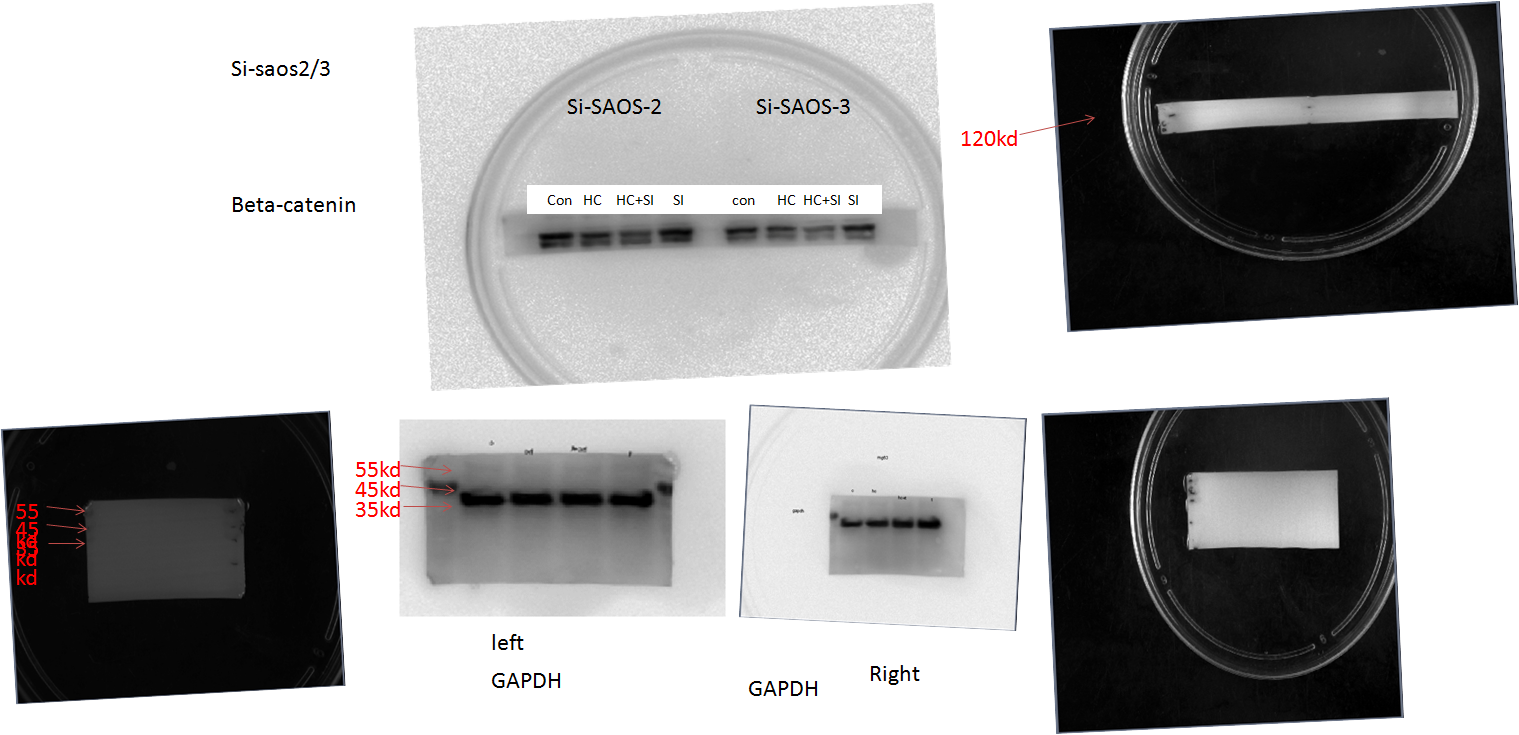

Supplement: Supplementary file 7 — Additional file 7. [file 40360_2023_655_MOESM7_ESM.zip › fig 7original/4group╠ß╜╗/Saos╠ß╜╗/beta-catenin/2.tif]

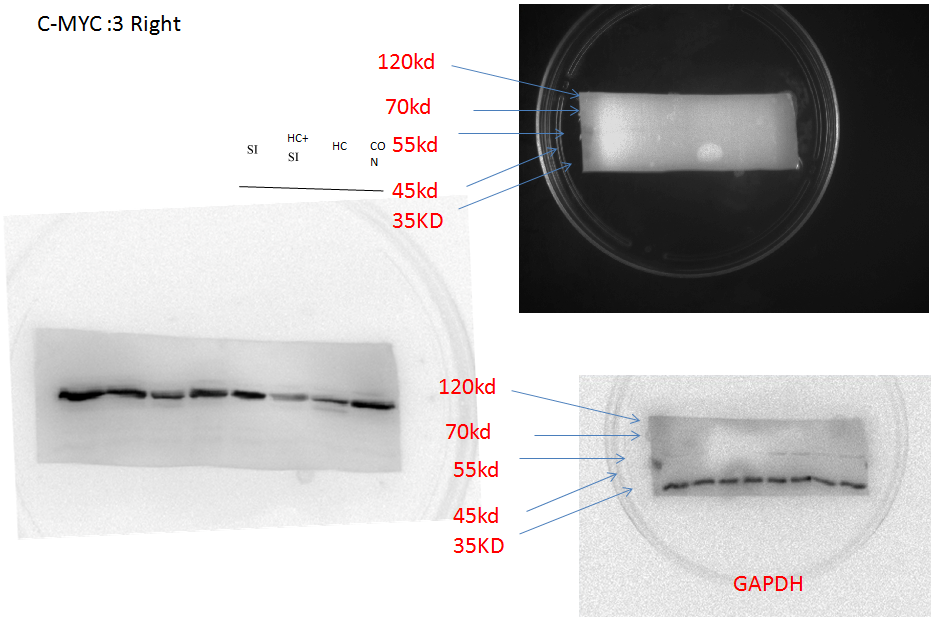

Supplement: Supplementary file 7 — Additional file 7. [file 40360_2023_655_MOESM7_ESM.zip › fig 7original/4group╠ß╜╗/Saos╠ß╜╗/cmyc/1.tif]

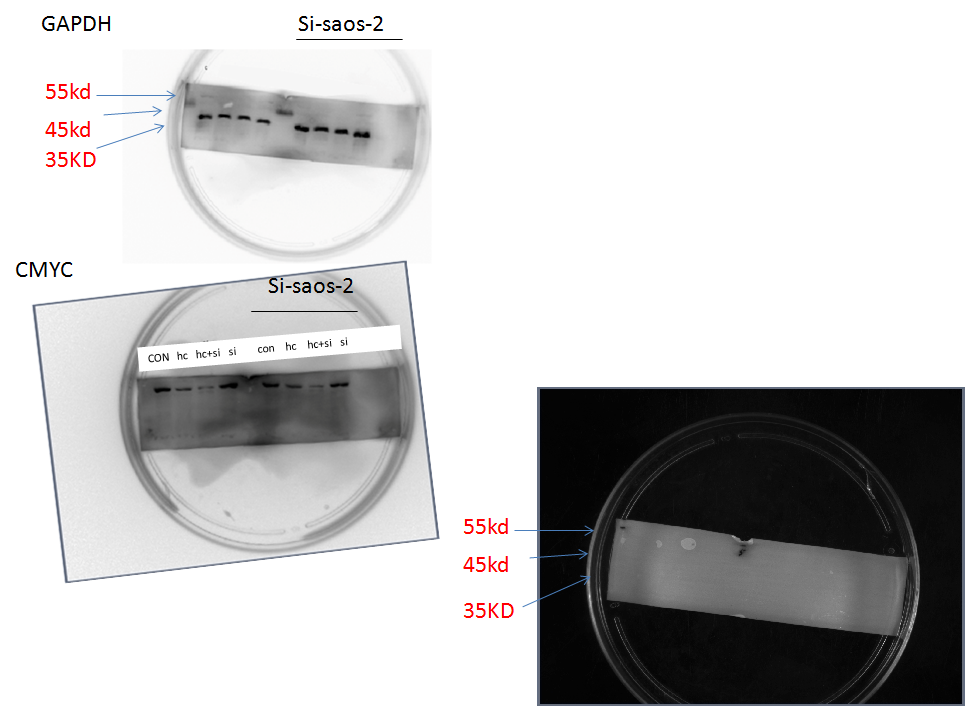

Supplement: Supplementary file 7 — Additional file 7. [file 40360_2023_655_MOESM7_ESM.zip › fig 7original/4group╠ß╜╗/Saos╠ß╜╗/cmyc/2.tif]

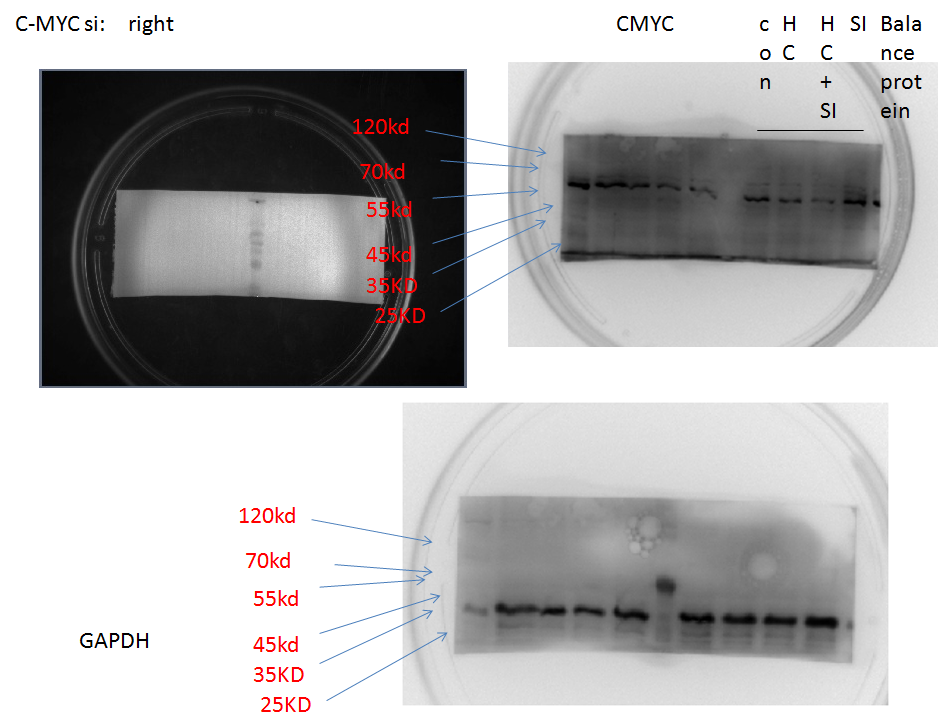

Supplement: Supplementary file 7 — Additional file 7. [file 40360_2023_655_MOESM7_ESM.zip › fig 7original/4group╠ß╜╗/Saos╠ß╜╗/cmyc/3.tif]

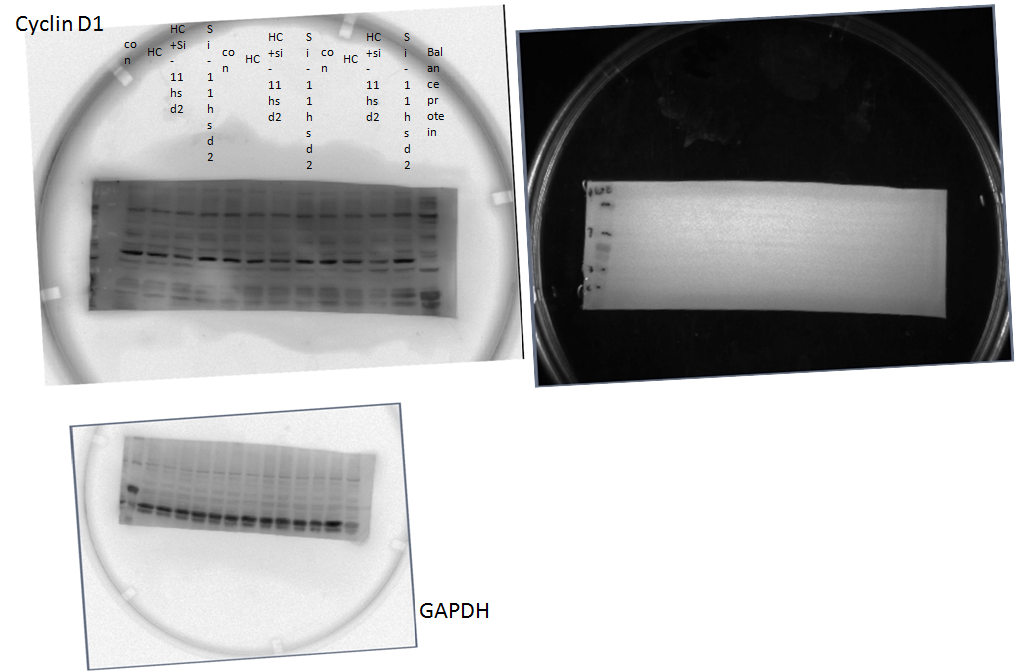

Supplement: Supplementary file 7 — Additional file 7. [file 40360_2023_655_MOESM7_ESM.zip › fig 7original/4group╠ß╜╗/Saos╠ß╜╗/cyclinD1/1.tif]

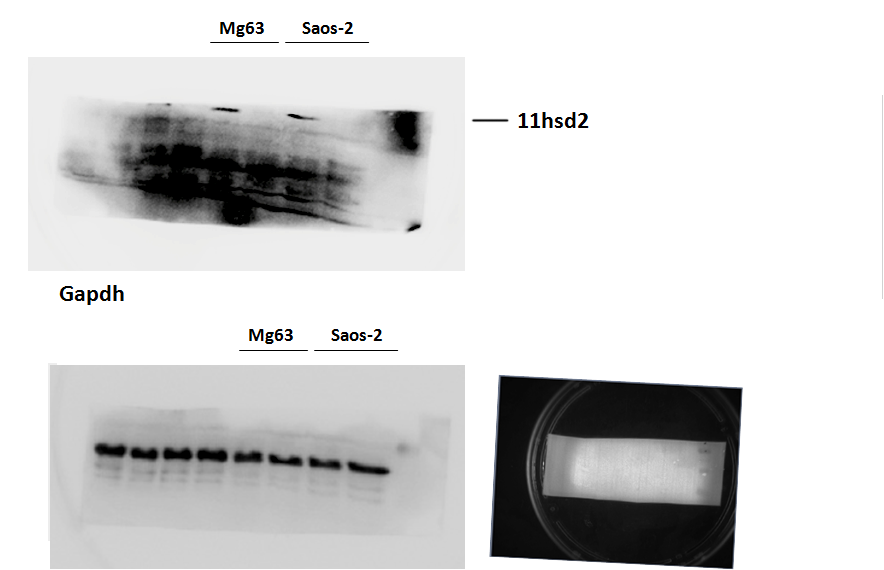

Supplement: Supplementary file 7 — Additional file 7. [file 40360_2023_655_MOESM7_ESM.zip › fig 7original/si11hsd2/1.tif]

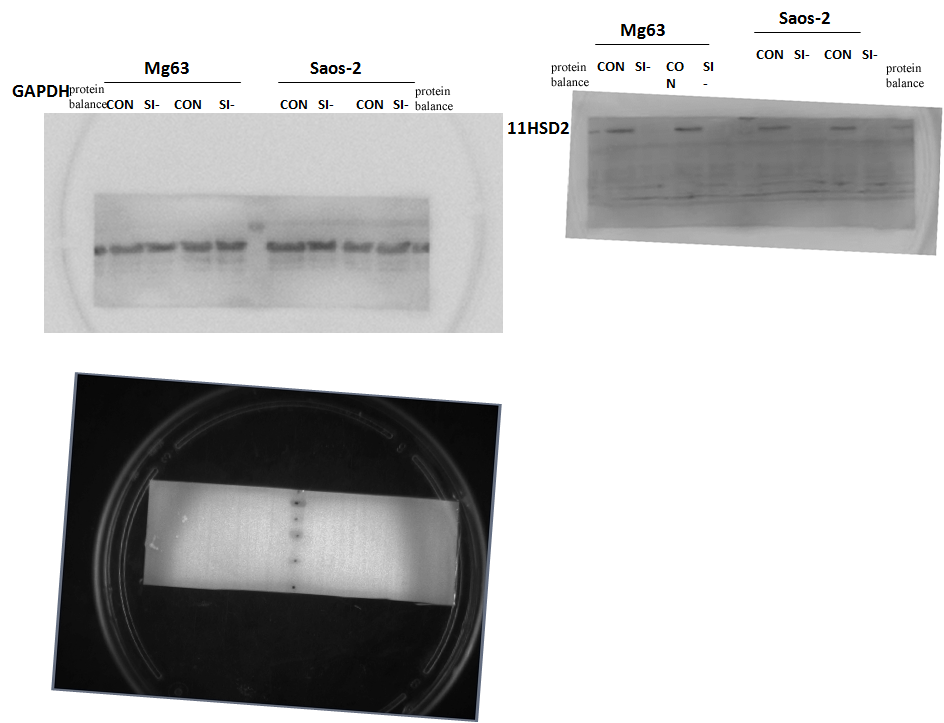

Supplement: Supplementary file 7 — Additional file 7. [file 40360_2023_655_MOESM7_ESM.zip › fig 7original/si11hsd2/2.tif]
