# Supplementary material for: Thiram, an inhibitor of 11ß-hydroxysteroid dehydrogenase type 2, enhances the inhibitory effects of hydrocortisone in the treatment of osteosarcoma through Wnt/β-catenin pathway
Source: BMC Pharmacol Toxicol. 2023 Mar 28;24:20. doi: 10.1186/s40360-023-00655-0 (PMC10045229; doi:10.1186/s40360-023-00655-0)
Supplement: Supplementary file 2 — Additional file 2. [file 40360_2023_655_MOESM2_ESM.docx]

1、c-MYC


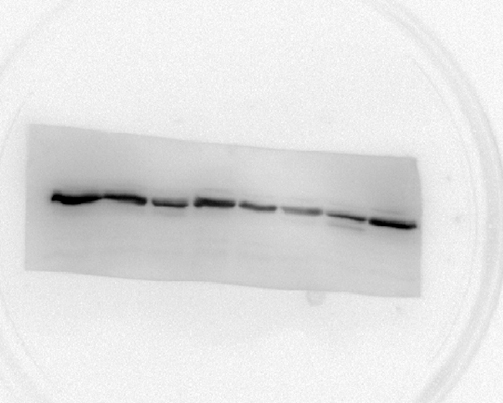


Control HC HC+thiram thiram

2、cyclin D1:


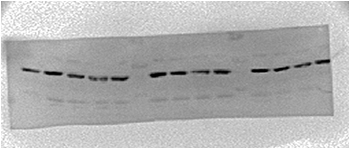


Control HC HC+thiram thiram

3. beta-catenin:


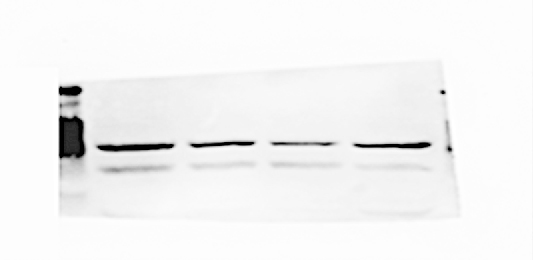


Control HC HC+thiram thiram

4. GAPDH:


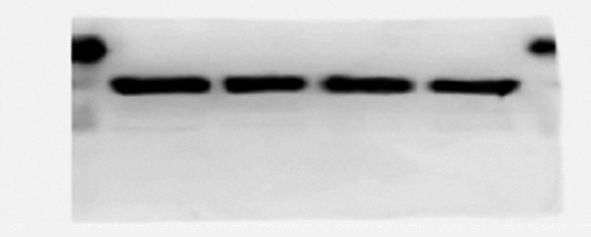


Control HC HC+thiram thiram
