# Supplementary material for: Thiram, an inhibitor of 11ß-hydroxysteroid dehydrogenase type 2, enhances the inhibitory effects of hydrocortisone in the treatment of osteosarcoma through Wnt/β-catenin pathway
Source: BMC Pharmacol Toxicol. 2023 Mar 28;24:20. doi: 10.1186/s40360-023-00655-0 (PMC10045229; doi:10.1186/s40360-023-00655-0)
Supplement: Supplementary file 3 — Additional file 3. [file 40360_2023_655_MOESM3_ESM.docx]

**1. c-MYC**


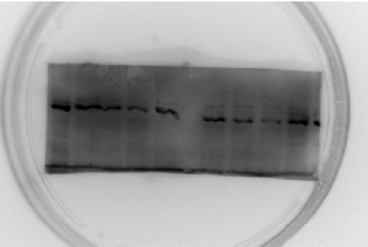


control

HC

HC+thiram

thiram

Balance protein

**2. Cyclin D1**





control

HC

HC+thiram

thiram

**3. beta-catenin**

**
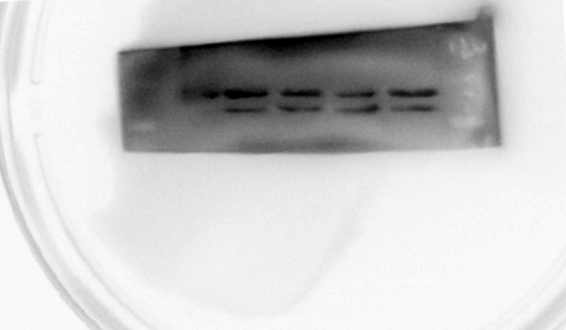
**

control

HC

HC+thiram

thiram

Balance protein

**4. GAPDH**

**
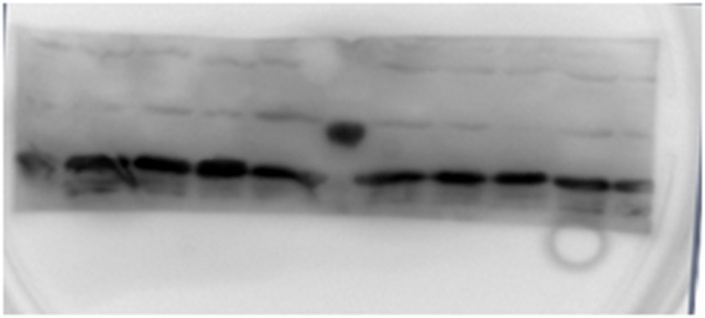
**

Balance protein

control

HC

HC+thiram

thiram
