# Supplementary material for: Thiram, an inhibitor of 11ß-hydroxysteroid dehydrogenase type 2, enhances the inhibitory effects of hydrocortisone in the treatment of osteosarcoma through Wnt/β-catenin pathway
Source: BMC Pharmacol Toxicol. 2023 Mar 28;24:20. doi: 10.1186/s40360-023-00655-0 (PMC10045229; doi:10.1186/s40360-023-00655-0)
Supplement: Supplementary file 6 — Additional file 6. [file 40360_2023_655_MOESM6_ESM.docx]

**1. 11HSD2：**





**Mg63 saos2**

**GAPDH of 11HSD2：**


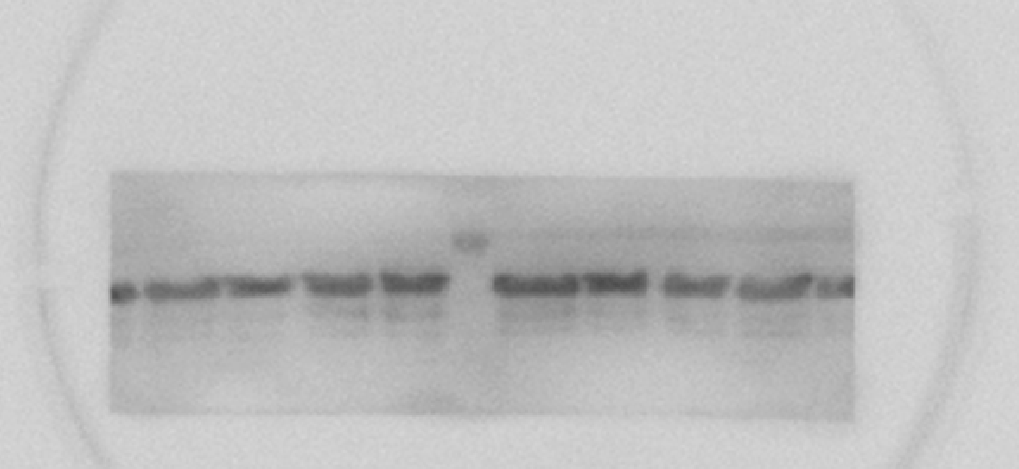


**2. CMYC**

①saos2：


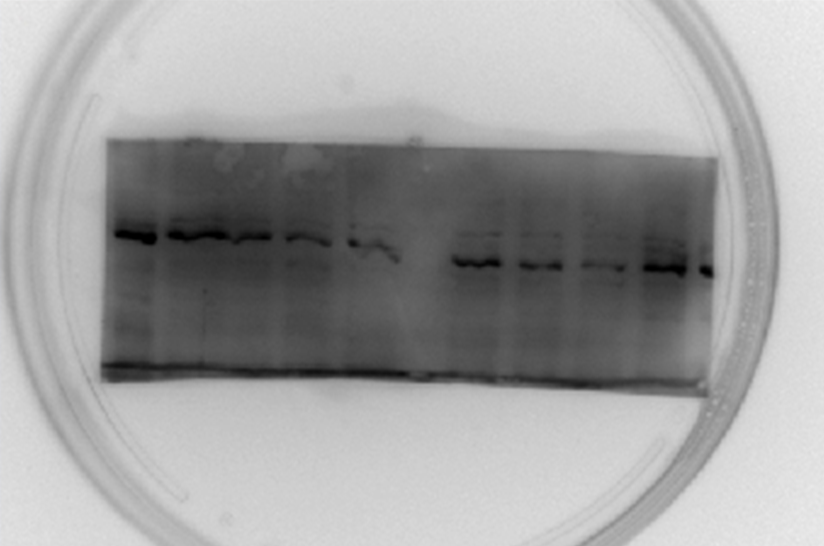


Con HC HC+SI SI

②Mg63：


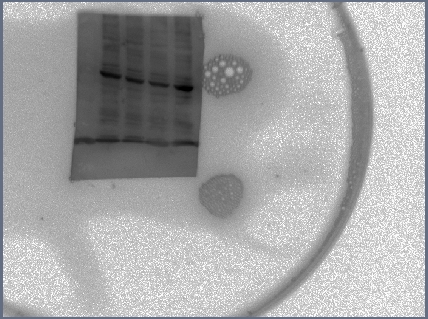


**3、CYCLIN：**

①saos2


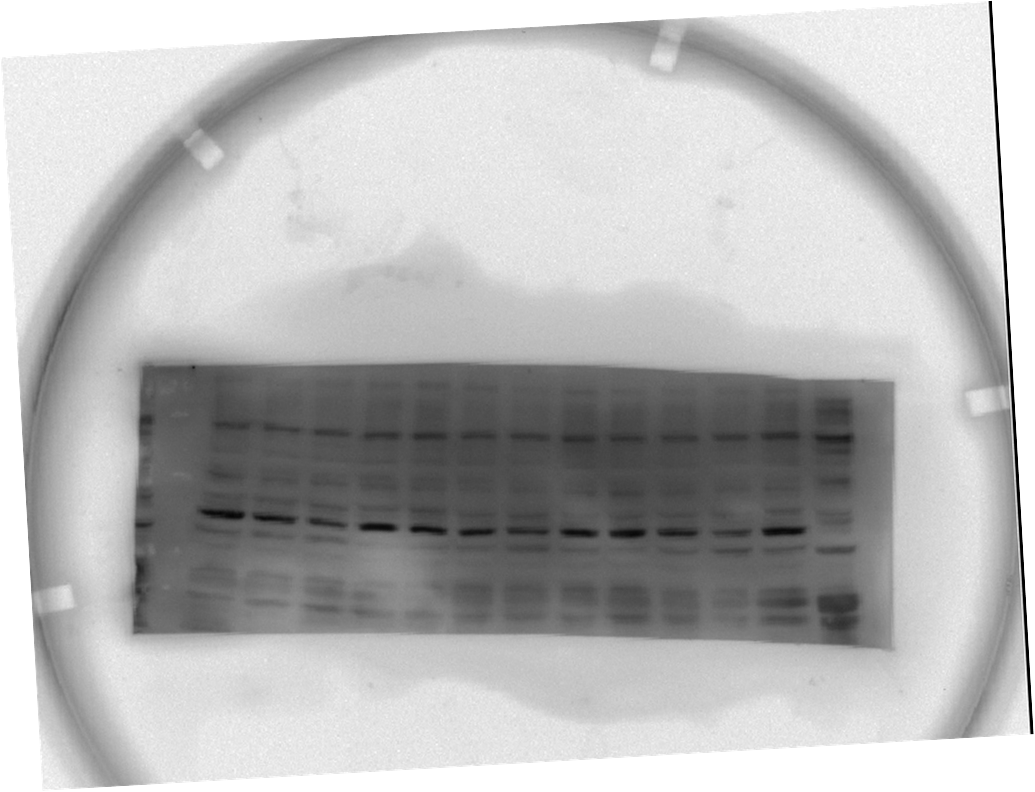


Con HC HC+SI SI

②Mg63:

**
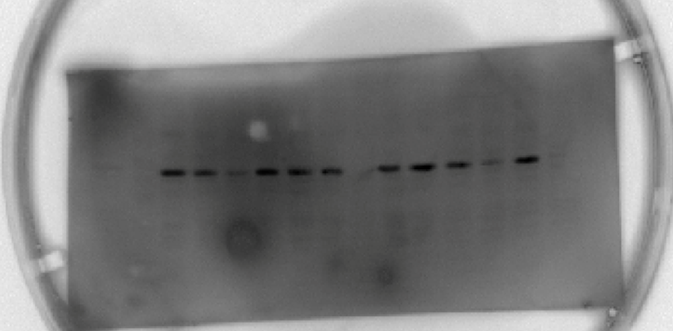
**

Con HC HC+SI SI

**4. beta-catenin:**

①saos2:


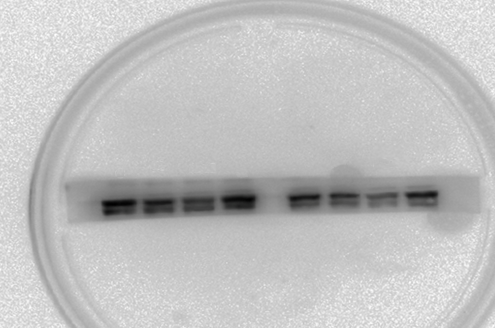


Con HC HC+SI SI

②Mg63:

Control HC HC+SI SI

**
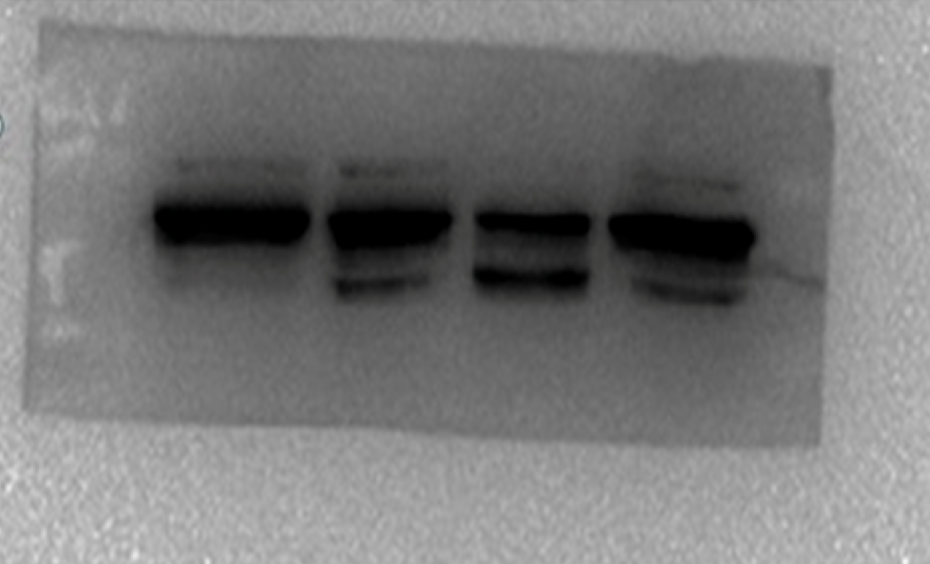
**

**5.GAPDH**

①saos2:

Con HC HC+SI SI

**
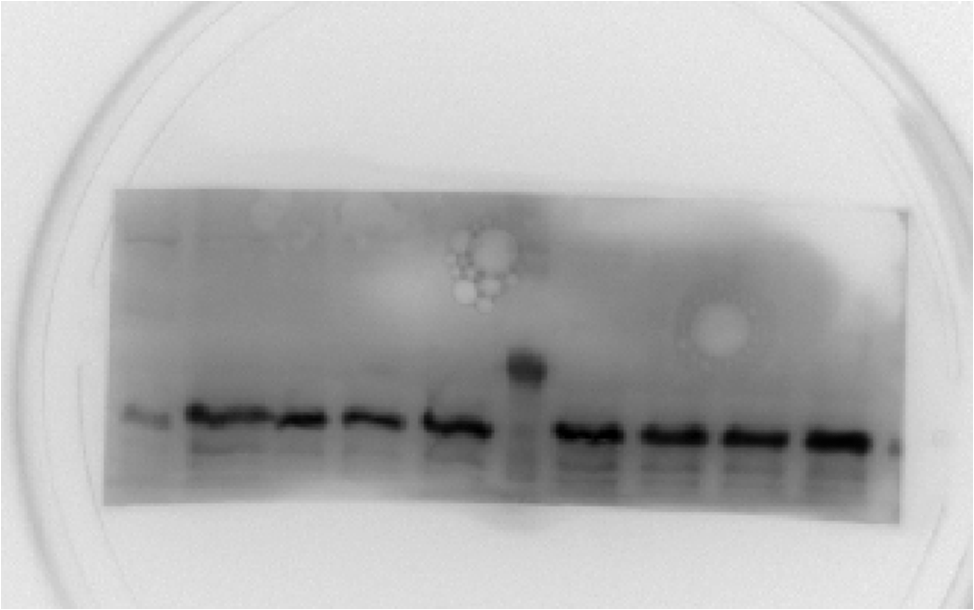
**

**②Mg63:**

Con HC HC+SI SI

**
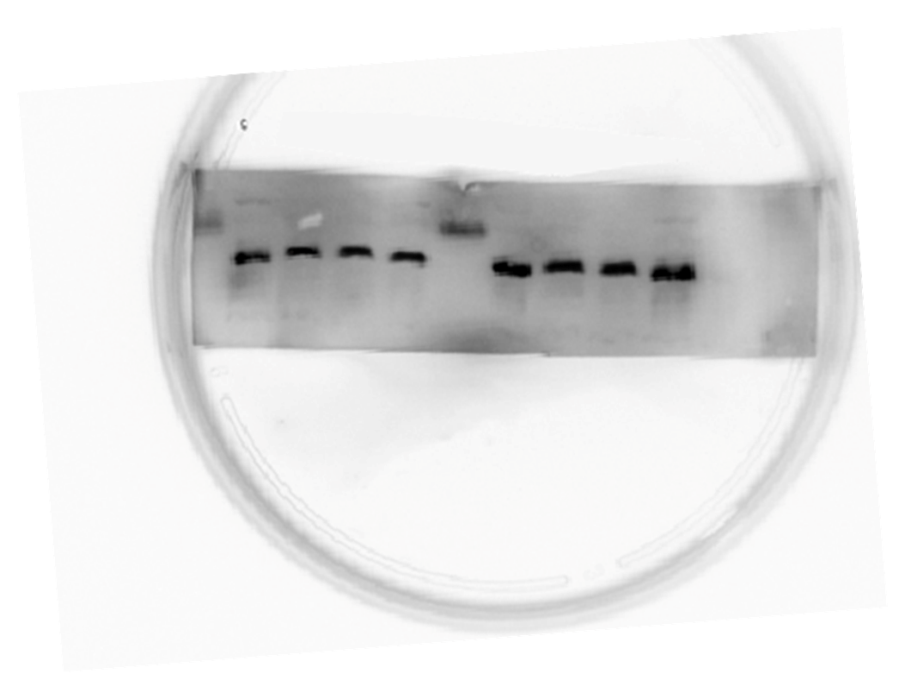
**
